# Supplementary material for: Multiple drivers and lineage-specific insect extinctions during the Permo–Triassic
Source: Nat Commun. 2022 Dec 6;13:7512. doi: 10.1038/s41467-022-35284-4 (PMC9726944; doi:10.1038/s41467-022-35284-4)
Supplement: Supplementary file 1 — Supplementary Information [file 41467_2022_35284_MOESM1_ESM.pdf]

Supplementary Information for

Multi-drivers and lineage-specific insect extinctions during the Permo–Triassic

Corentin Jouault, André Nel, Vincent Perrichot, Frédéric Legendre, Fabien L. Condamine

Corresponding authors: Corentin Jouault and Fabien L. Condamine

Emails: jouaultc0@gmail.com / fabien.condamine@gmail.com

This PDF file includes:

Supplementary Figures 1 to 24

Supplementary Tables 1 to 14

Supplementary Figure 1. Frequency of rate shifts for origination and extinction for all insects. At the genus (A and B), and family (C and D) level as inferred by PyRate using reversible jump Markov Chain Monte Carlo. Origination (A and C) and extinction rates (B and D) significance is evidenced with horizontal dashed lines indicating log-Bayes factors of 2 (bottom) and 6 (top). Sampling frequencies higher than log-Bayes factors = 6 indicate strong statistical support for a rate shift. Solid lines indicate mean posterior rates and the shaded areas show 95% credibility intervals. The red vertical line indicates the Permian-Triassic boundary. First orange period represents the GEE, the second is the CPE. Time is in millions of years. The color of each period in the chronostratigraphic scale follows that of the International Chronostratigraphic Chart (v2022/02).

Supplementary Figure 1.

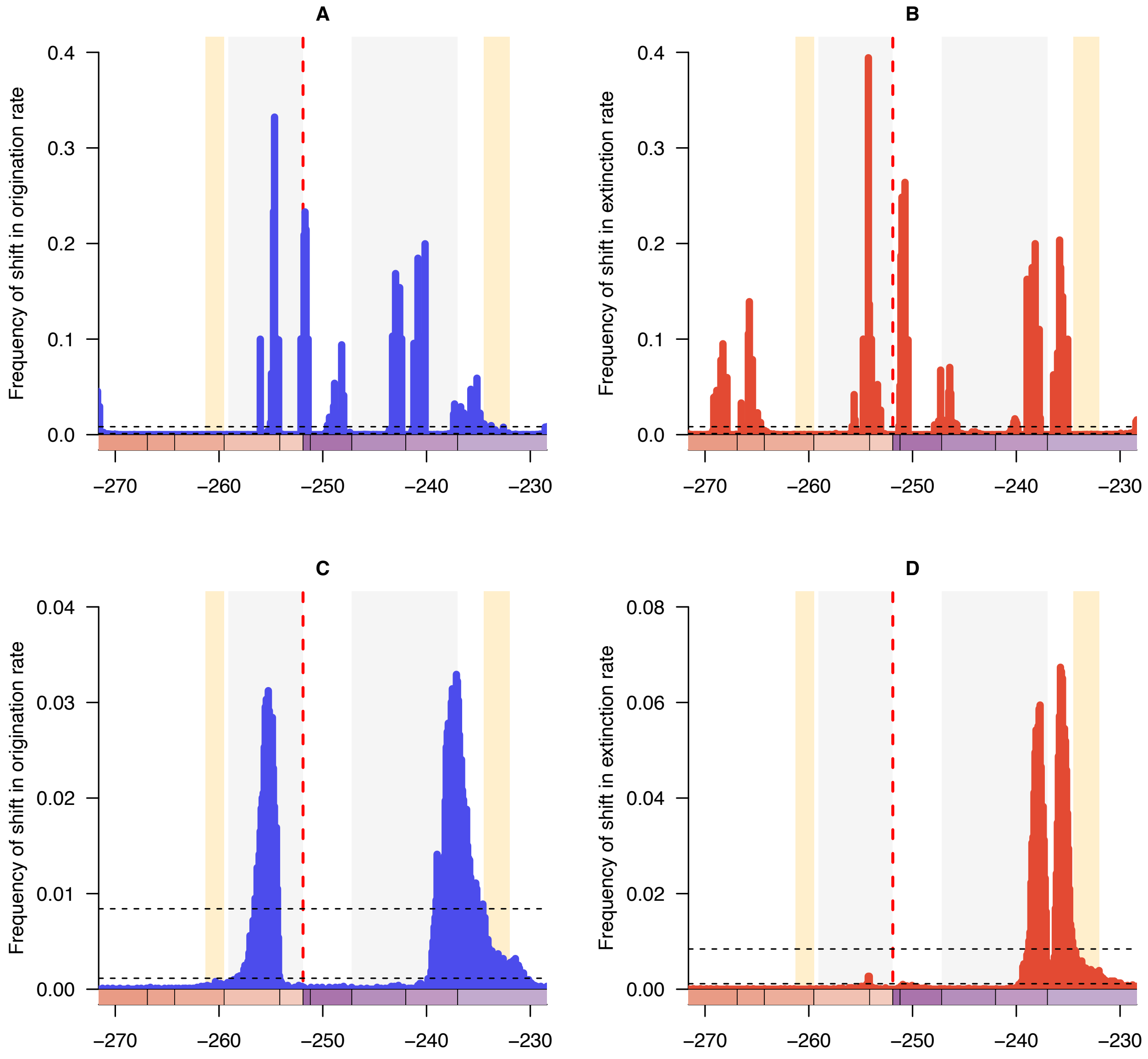

Supplementary Figure 2. Frequency of rate shifts for origination and extinction for the major clades of insects. At the genus level as inferred by PyRate using reversible jump Markov Chain Monte Carlo. Shifts are detailed for Polyneoptera (A and B), Holometabola (C and D), Acercaria (E and F), and Paleoptera (G and H). Origination (A, C, E, and G) and extinction rates (B, D, F and H) significance is evidenced with horizontal dashed lines indicating log-Bayes factors of 2 (bottom) and 6 (top). Sampling frequencies higher than log-Bayes factors = 6 indicate strong statistical support for a rate shift. Solid lines indicate mean posterior rates and the shaded areas show 95% credibility intervals. First orange period represents the GEE, the second is the CPE. Time is in millions of years. The color of each period in the chronostratigraphic scale follows that of the International Chronostratigraphic Chart (v2022/02).

Supplementary Figure 2.

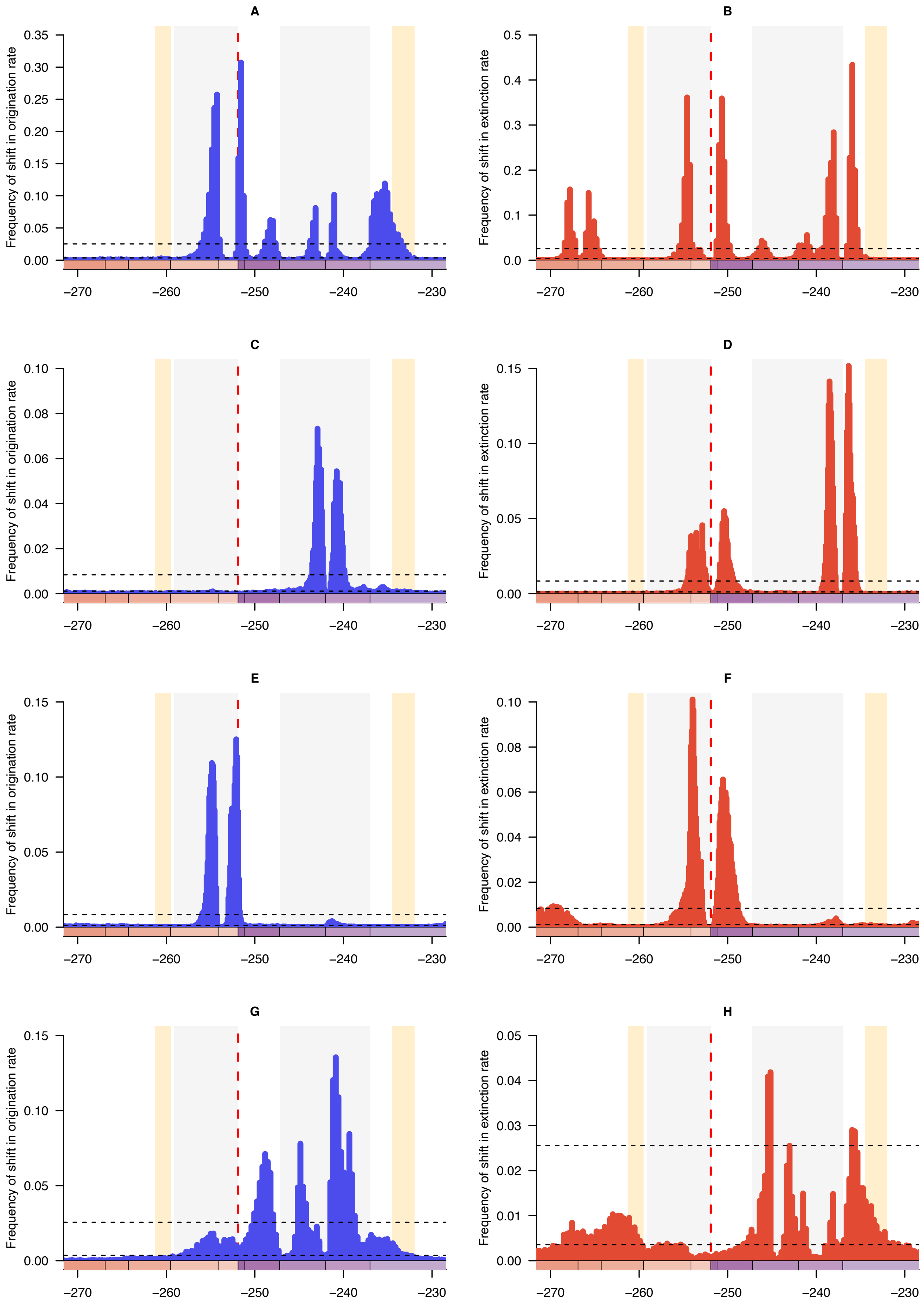

Supplementary Figure 3. Bayesian fossil-based inferences of insect origination (A) and extinction (B) rates at the genus level under the birth-death model with stages as constrained shifts. (C) The net diversification rates are obtained with the difference between origination and extinction rates (rates below 0 indicate declining diversity). Solid lines indicate mean posterior rates and the shaded areas show 95% credibility intervals. First orange period represents the GEE, the second is the CPE. Time is in millions of years. The color of each period in the chronostratigraphic scale follows that of the International Chronostratigraphic Chart (v2022/02).

Supplementary Figure 3.

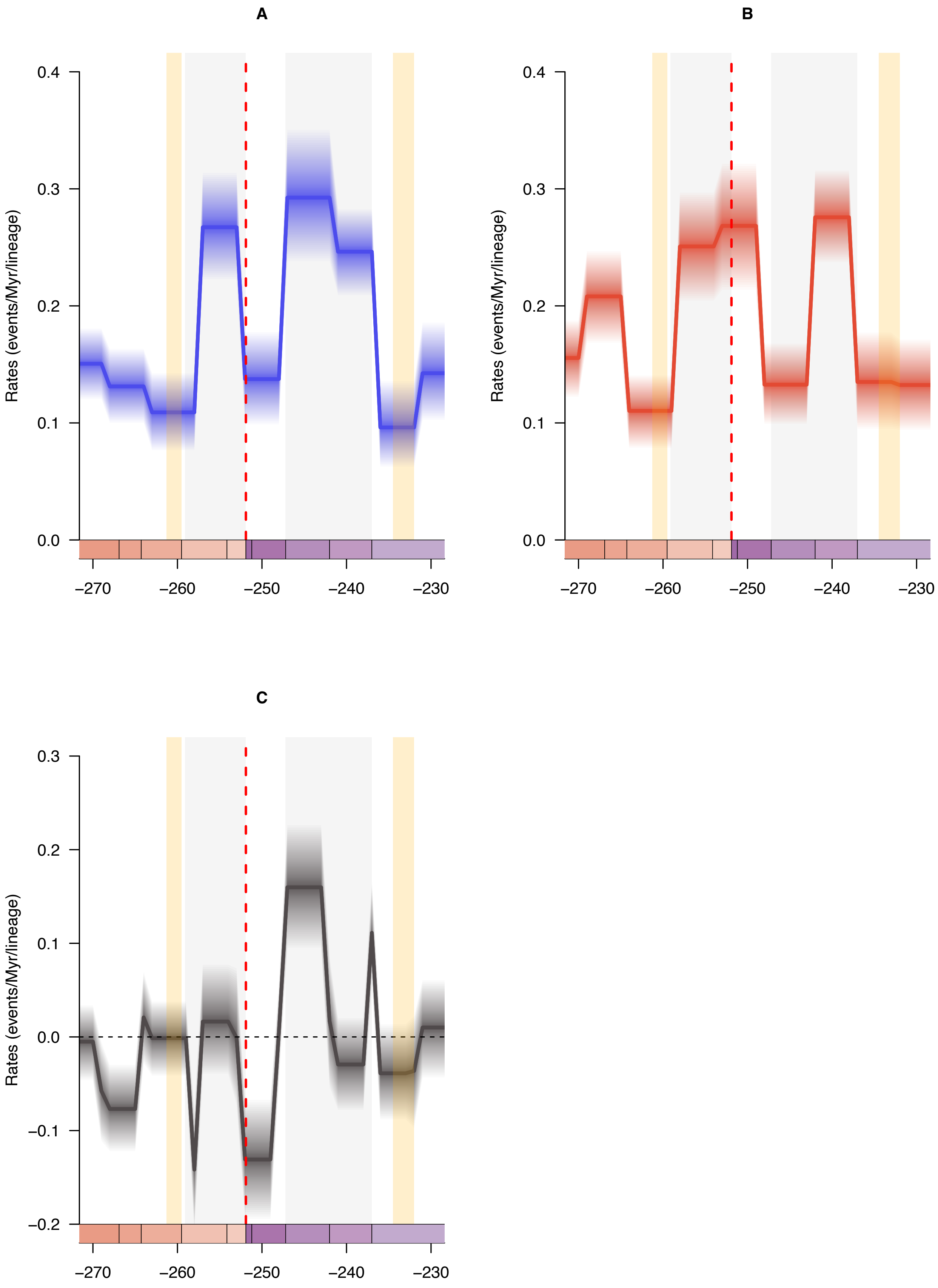

Supplementary Figure 4. Bayesian fossil-based inferences of insect origination (A) and extinction (B) rates at the family level under the birth-death model with stages as constrained shifts. (C) The net diversification rates are obtained with the difference between origination and extinction rates (rates below 0 indicate declining diversity). Solid lines indicate mean posterior rates and the shaded areas show 95% credibility intervals. First orange period represents the GEE, the second is the CPE. Time is in millions of years. The color of each period in the chronostratigraphic scale follows that of the International Chronostratigraphic Chart (v2022/02).

Supplementary Figure 4.

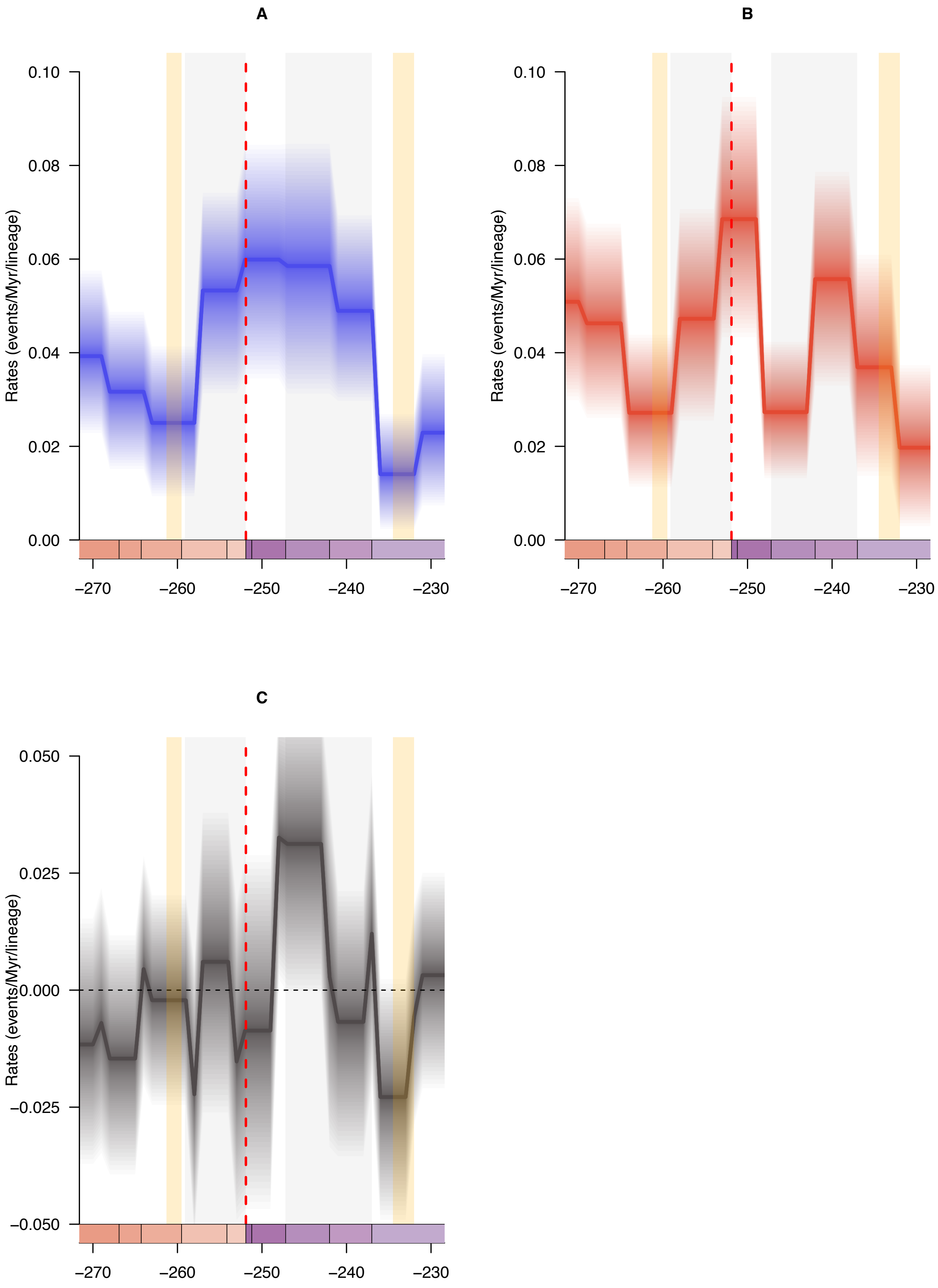

Supplementary Figure 5. Diversification and diversity dynamics of all Coleoptera genera. Bayesian estimations of origination and extinction rates through time as inferred by PyRate using reversible jump Markov Chain Monte Carlo. Marginal estimates of origination rates (A) and extinction rates (C) through time are shown as mean and 95% credibility intervals (shaded areas). The frequency of a sampled rate shift is computed within small time bins for origination and extinction rates (B and D, respectively), with horizontal dashed lines indicating log-Bayes factors of 2 (bottom) and 6 (top). Sampling frequencies higher than log-Bayes factors = 6 indicate strong statistical support for a rate shift. Panel (E) shows net diversification rates through time (computed as the posterior difference between origination and extinction rates through time). Panel (F) shows the number of genera through time computed by summing up the lifespans of all genus. For each plot, solid lines indicate mean posterior rates; shaded areas show 95% CI. The red vertical line indicates the Permian-Triassic boundary. First orange period represents the GEE, the second is the CPE. Time is in millions of years. The color of each period in the chronostratigraphic scale follows that of the International Chronostratigraphic Chart (v2022/02).

Supplementary Figure 5.

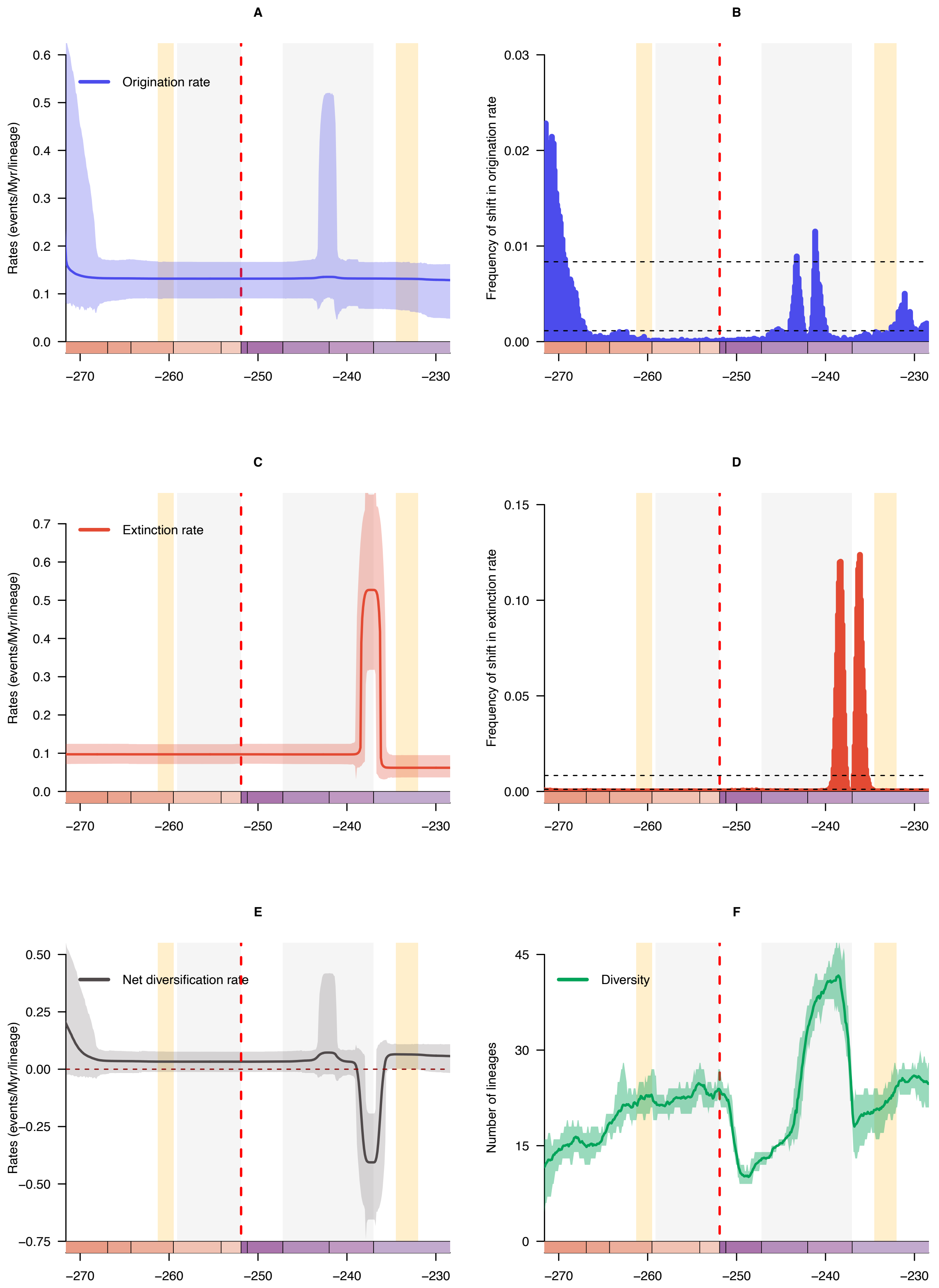

Supplementary Figure 6. Diversification and diversity dynamics of all insect genera without singleton. Bayesian estimations of origination and extinction rates through time as inferred by PyRate using reversible jump Markov Chain Monte Carlo. Marginal estimates of origination rates (A) and extinction rates (C) through time are shown as mean and 95% credibility intervals (shaded areas). The frequency of a sampled rate shift is computed within small time bins for origination and extinction rates (B and D, respectively), with horizontal dashed lines indicating log-Bayes factors of 2 (bottom) and 6 (top). Sampling frequencies higher than log-Bayes factors = 6 indicate strong statistical support for a rate shift. Panel (E) shows net diversification rates through time (computed as the posterior difference between origination and extinction rates through time). Panel (F) shows the number of genera through time computed by summing up the lifespans of all genus. For each plot, solid lines indicate mean posterior rates; shaded areas show 95% CI. The red vertical line indicates the Permian-Triassic boundary. First orange period represents the GEE, the second is the CPE. Time is in millions of years. The color of each period in the chronostratigraphic scale follows that of the International Chronostratigraphic Chart (v2022/02).

Supplementary Figure 6.

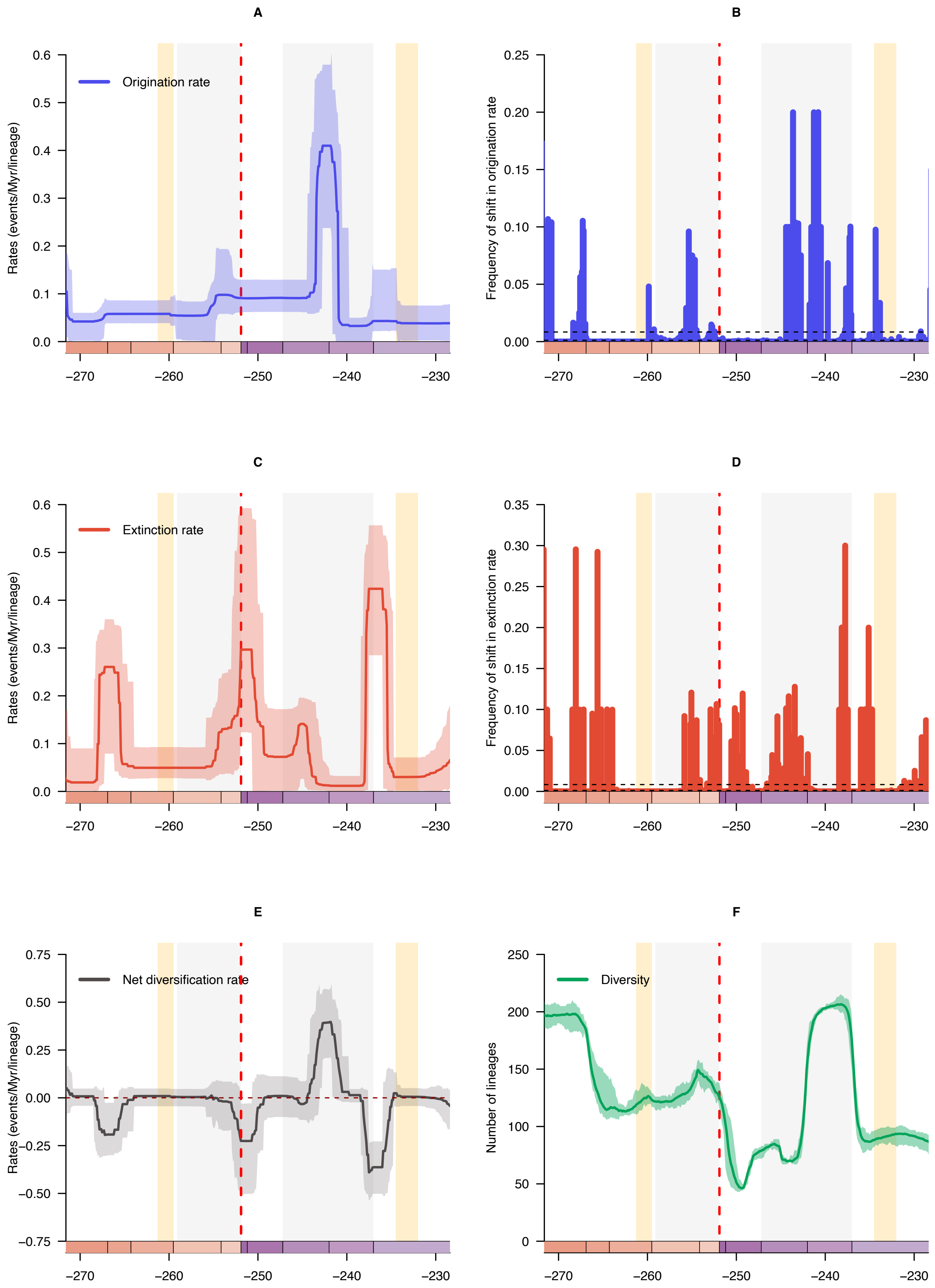

Supplementary Figure 7. Diversification and diversity dynamics of all Mecoptera genera. Bayesian estimations of origination and extinction rates through time as inferred by PyRate using reversible jump Markov Chain Monte Carlo. Marginal estimates of origination rates (A) and extinction rates (C) through time are shown as mean and 95% credibility intervals (shaded areas). The frequency of a sampled rate shift is computed within small time bins for origination and extinction rates (B and D, respectively), with horizontal dashed lines indicating log-Bayes factors of 2 (bottom) and 6 (top). Sampling frequencies higher than log-Bayes factors = 6 indicate strong statistical support for a rate shift. Panel (E) shows net diversification rates through time (computed as the posterior difference between origination and extinction rates through time). Panel (F) shows the number of genera through time computed by summing up the lifespans of all genus. For each plot, solid lines indicate mean posterior rates; shaded areas show 95% CI. The red vertical line indicates the Permian-Triassic boundary. First orange period represents the GEE, the second is the CPE. Time is in millions of years. The color of each period in the chronostratigraphic scale follows that of the International Chronostratigraphic Chart (v2022/02).

Supplementary Figure 7.

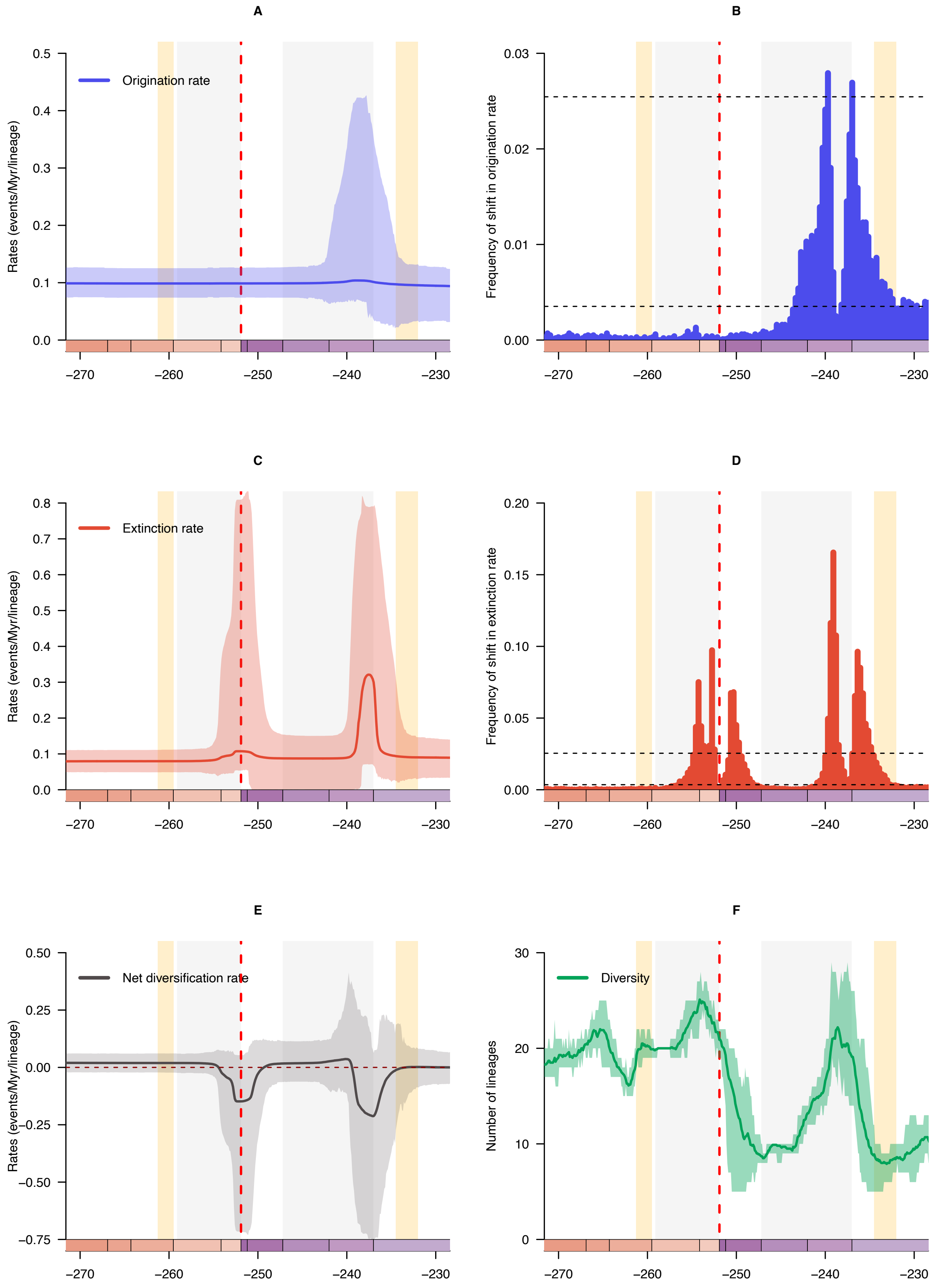

Supplementary Figure 8. Diversification and diversity dynamics of all Hemiptera genera. Bayesian estimations of origination and extinction rates through time as inferred by PyRate using reversible jump Markov Chain Monte Carlo. Marginal estimates of origination rates (A) and extinction rates (C) through time are shown as mean and 95% credibility intervals (shaded areas). The frequency of a sampled rate shift is computed within small time bins for origination and extinction rates (B and D, respectively), with horizontal dashed lines indicating log-Bayes factors of 2 (bottom) and 6 (top). Sampling frequencies higher than log-Bayes factors = 6 indicate strong statistical support for a rate shift. Panel (E) shows net diversification rates through time (computed as the posterior difference between origination and extinction rates through time). Panel (F) shows the number of genera through time computed by summing up the lifespans of all genus. For each plot, solid lines indicate mean posterior rates; shaded areas show 95% CI. The red vertical line indicates the Permian-Triassic boundary. First orange period represents the GEE, the second is the CPE. Time is in millions of years. The color of each period in the chronostratigraphic scale follows that of the International Chronostratigraphic Chart (v2022/02).

Supplementary Figure 8.

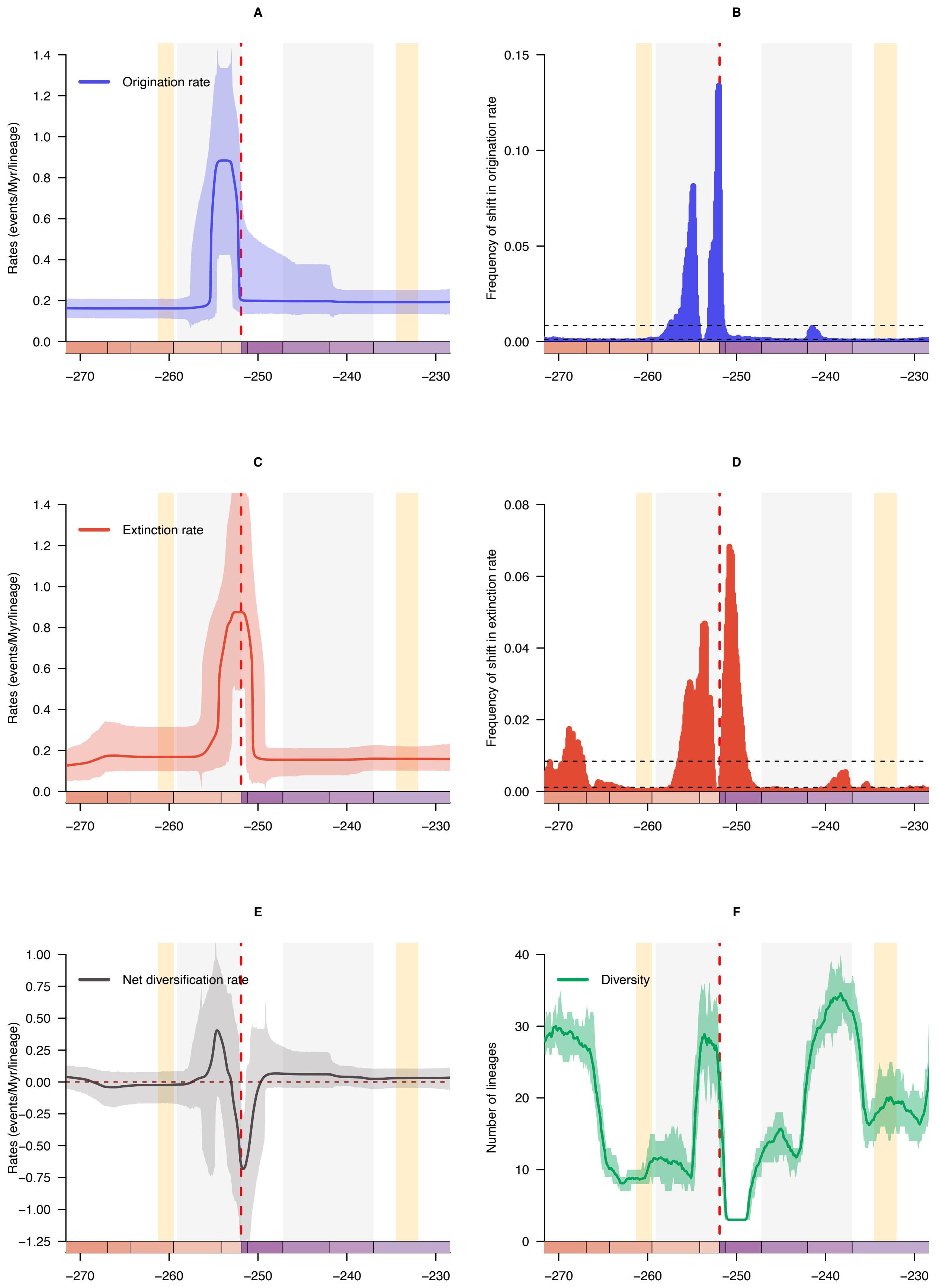

Supplementary Figure 9. Diversification and diversity dynamics of all Orthoptera and Titanoptera genera. Bayesian estimations of origination and extinction rates through time as inferred by PyRate using reversible jump Markov Chain Monte Carlo. Marginal estimates of origination rates (A) and extinction rates (C) through time are shown as mean and 95% credibility intervals (shaded areas). The frequency of a sampled rate shift is computed within small time bins for origination and extinction rates (B and D, respectively), with horizontal dashed lines indicating log-Bayes factors of 2 (bottom) and 6 (top). Sampling frequencies higher than log-Bayes factors = 6 indicate strong statistical support for a rate shift. Panel (E) shows net diversification rates through time (computed as the posterior difference between origination and extinction rates through time). Panel (F) shows the number of genera through time computed by summing up the lifespans of all genus. For each plot, solid lines indicate mean posterior rates; shaded areas show 95% CI. The red vertical line indicates the Permian-Triassic boundary. First orange period represents the GEE, the second is the CPE. Time is in millions of years. The color of each period in the chronostratigraphic scale follows that of the International Chronostratigraphic Chart (v2022/02).

Supplementary Figure 9.

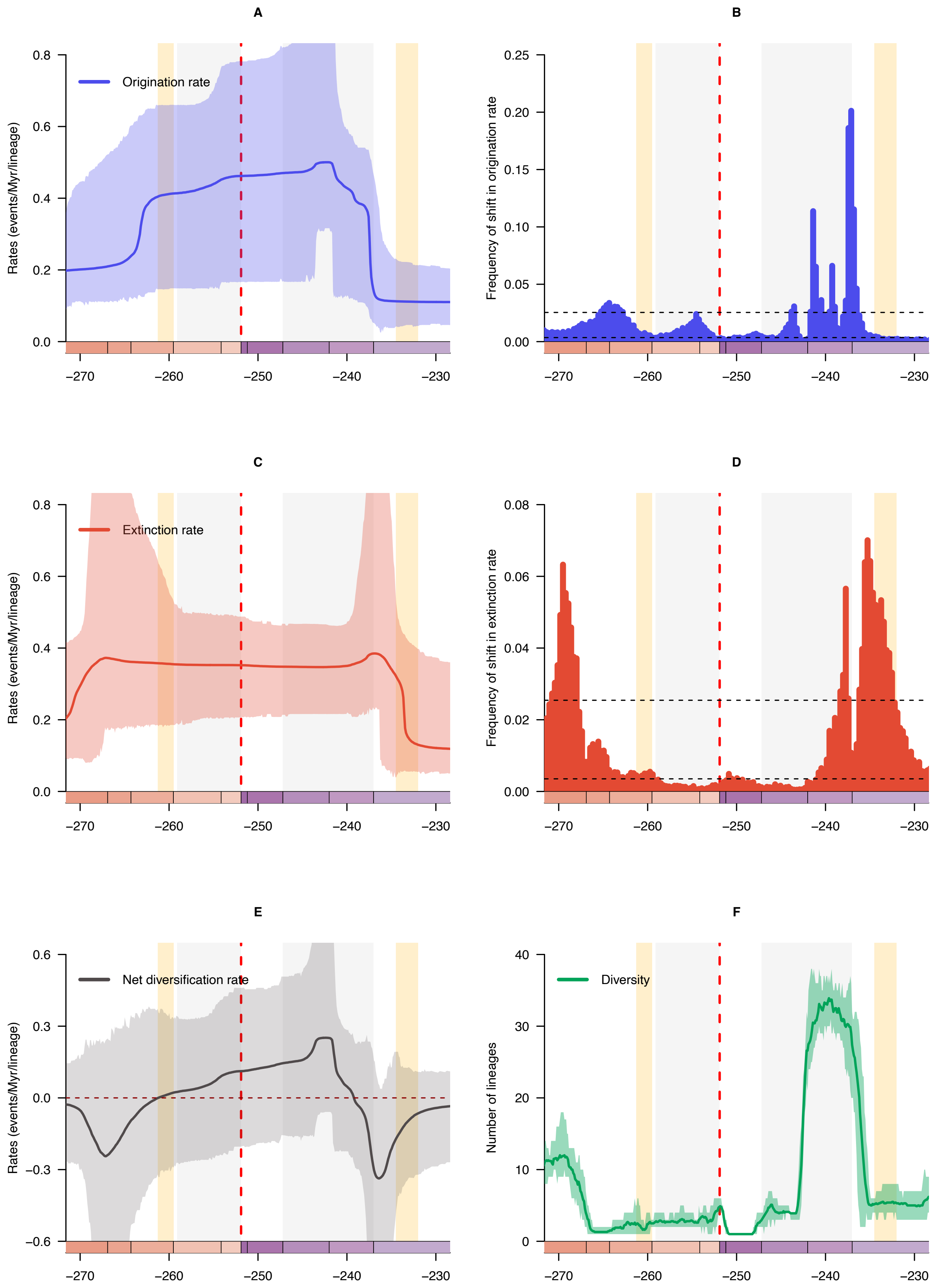

Supplementary Figure 10. Diversification and diversity dynamics of all Odonatoptera genera. Bayesian estimations of origination and extinction rates through time as inferred by PyRate using reversible jump Markov Chain Monte Carlo. Marginal estimates of origination rates (A) and extinction rates (C) through time are shown as mean and 95% credibility intervals (shaded areas). The frequency of a sampled rate shift is computed within small time bins for origination and extinction rates (B and D, respectively), with horizontal dashed lines indicating log-Bayes factors of 2 (bottom) and 6 (top). Sampling frequencies higher than log-Bayes factors = 6 indicate strong statistical support for a rate shift. Panel (E) shows net diversification rates through time (computed as the posterior difference between origination and extinction rates through time). Panel (F) shows the number of genera through time computed by summing up the lifespans of all genus. For each plot, solid lines indicate mean posterior rates; shaded areas show 95% CI. The red vertical line indicates the Permian-Triassic boundary. First orange period represents the GEE, the second is the CPE. Time is in millions of years. The color of each period in the chronostratigraphic scale follows that of the International Chronostratigraphic Chart (v2022/02).

Supplementary Figure 10.

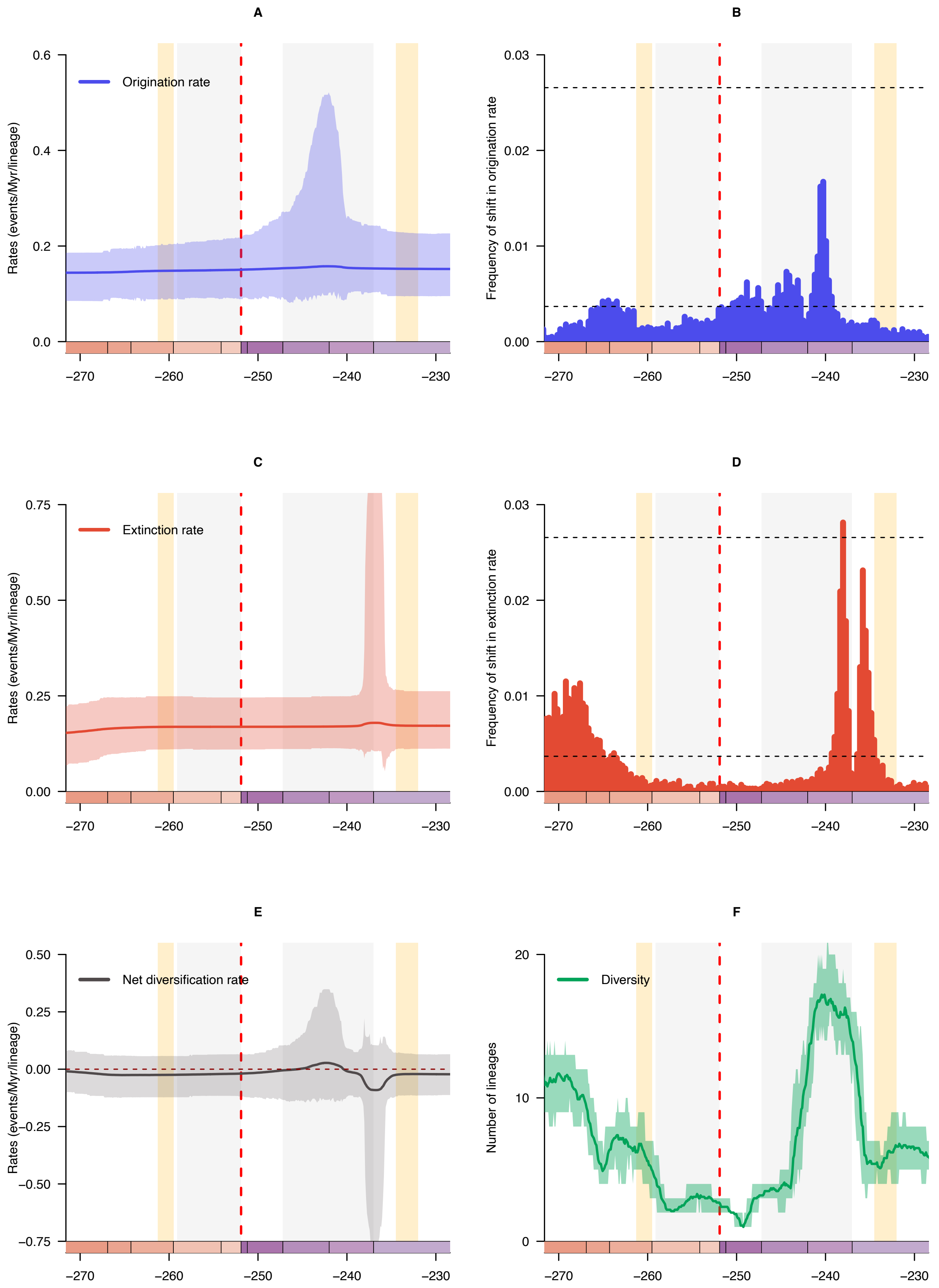

Supplementary Figure 11. Diversification and diversity dynamics of all Paleodictyopteroidea genera. Bayesian estimations of origination and extinction rates through time as inferred by PyRate using reversible jump Markov Chain Monte Carlo. Marginal estimates of origination rates (A) and extinction rates (C) through time are shown as mean and 95% credibility intervals (shaded areas). The frequency of a sampled rate shift is computed within small time bins for origination and extinction rates (B and D, respectively), with horizontal dashed lines indicating log-Bayes factors of 2 (bottom) and 6 (top). Sampling frequencies higher than log-Bayes factors = 6 indicate strong statistical support for a rate shift. Panel (E) shows net diversification rates through time (computed as the posterior difference between origination and extinction rates through time). Panel (F) shows the number of genera through time computed by summing up the lifespans of all genus. For each plot, solid lines indicate mean posterior rates; shaded areas show 95% CI. The red vertical line indicates the Permian-Triassic boundary. First orange period represents the GEE, the second is the CPE. Time is in millions of years. The color of each period in the chronostratigraphic scale follows that of the International Chronostratigraphic Chart (v2022/02).

Supplementary Figure 11.

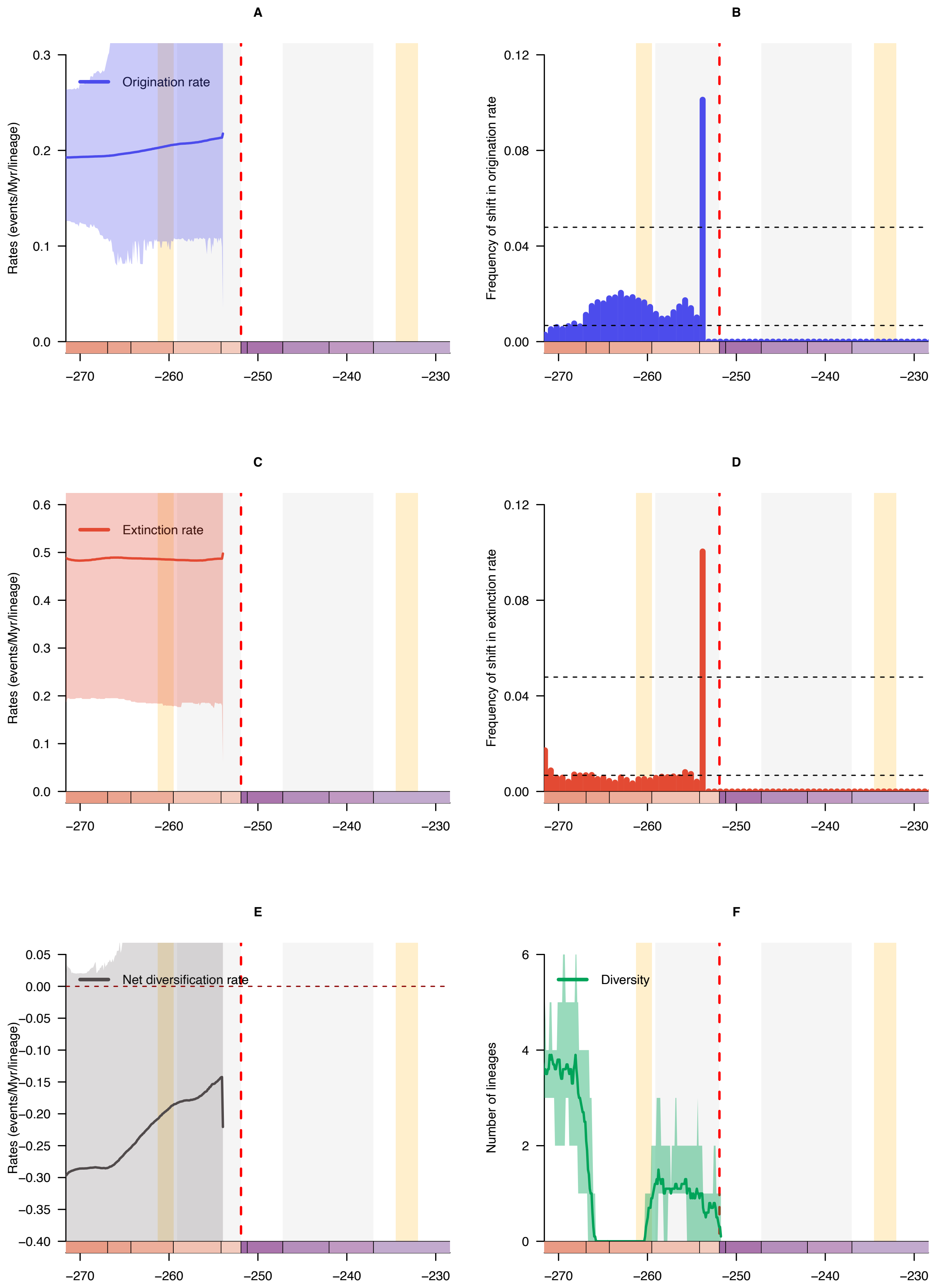

Supplementary Figure 12. Diversification and diversity dynamics of all Acercaria families. Bayesian estimations of origination and extinction rates through time as inferred by PyRate using reversible jump Markov Chain Monte Carlo. Marginal estimates of origination rates (A) and extinction rates (C) through time are shown as mean and 95% credibility intervals (shaded areas). The frequency of a sampled rate shift is computed within small time bins for origination and extinction rates (B and D, respectively), with horizontal dashed lines indicating log-Bayes factors of 2 (bottom) and 6 (top). Sampling frequencies higher than log-Bayes factors = 6 indicate strong statistical support for a rate shift. Panel (E) shows net diversification rates through time (computed as the posterior difference between origination and extinction rates through time). Panel (F) shows the number of families through time computed by summing up the lifespans of all genus. For each plot, solid lines indicate mean posterior rates; shaded areas show 95% CI. The red vertical line indicates the Permian-Triassic boundary. First orange period represents the GEE, the second is the CPE. Time is in millions of years. The color of each period in the chronostratigraphic scale follows that of the International Chronostratigraphic Chart (v2022/02).

Supplementary Figure 12.

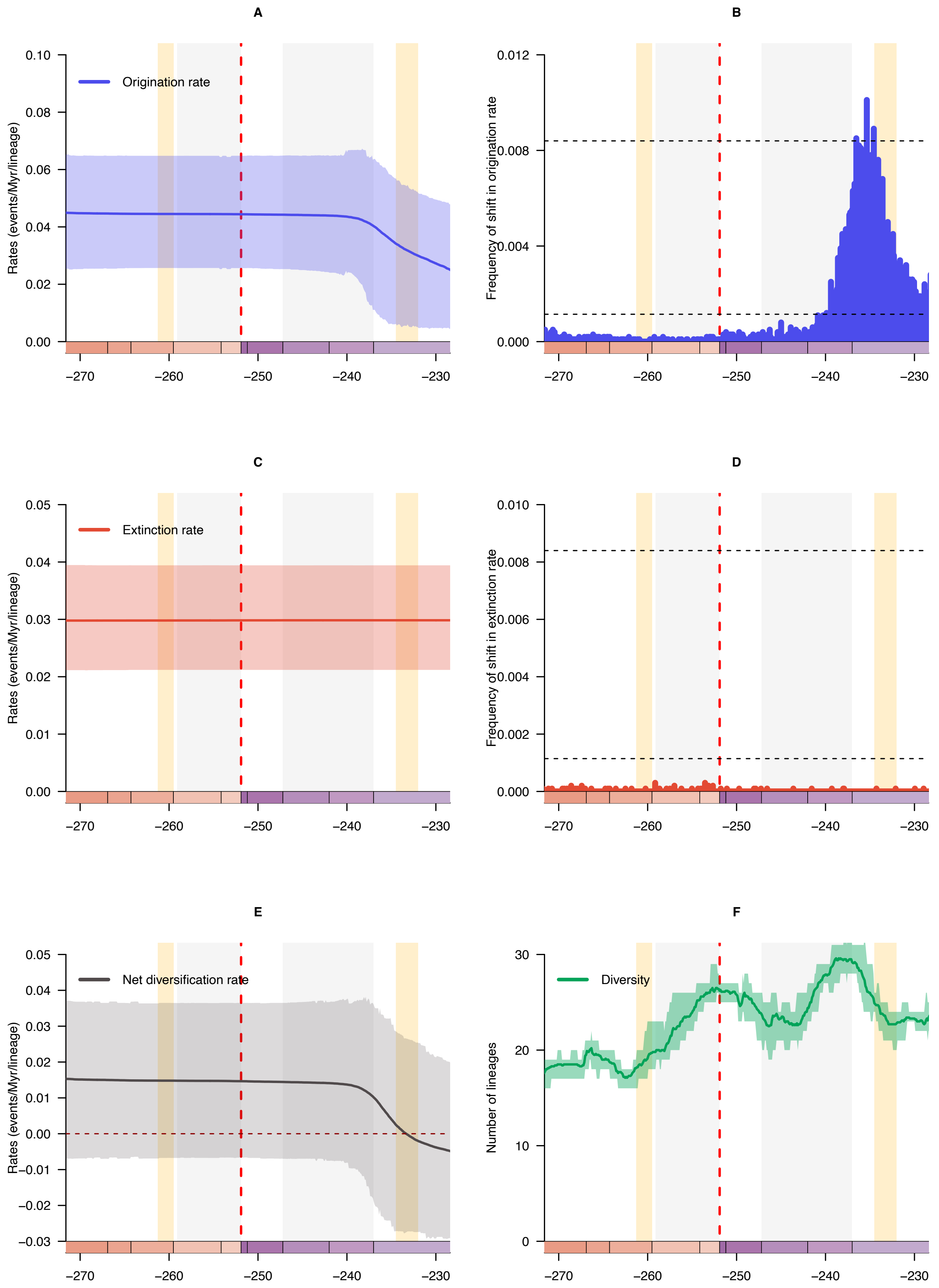

Supplementary Figure 13. Diversification and diversity dynamics of all Holometabola families. Bayesian estimations of origination and extinction rates through time as inferred by PyRate using reversible jump Markov Chain Monte Carlo. Marginal estimates of origination rates (A) and extinction rates (C) through time are shown as mean and 95% credibility intervals (shaded areas). The frequency of a sampled rate shift is computed within small time bins for origination and extinction rates (B and D, respectively), with horizontal dashed lines indicating log-Bayes factors of 2 (bottom) and 6 (top). Sampling frequencies higher than log-Bayes factors = 6 indicate strong statistical support for a rate shift. Panel (E) shows net diversification rates through time (computed as the posterior difference between origination and extinction rates through time). Panel (F) shows the number of families through time computed by summing up the lifespans of all genus. For each plot, solid lines indicate mean posterior rates; shaded areas show 95% CI. The red vertical line indicates the Permian-Triassic boundary. First orange period represents the GEE, the second is the CPE. Time is in millions of years. The color of each period in the chronostratigraphic scale follows that of the International Chronostratigraphic Chart (v2022/02).

Supplementary Figure 13.

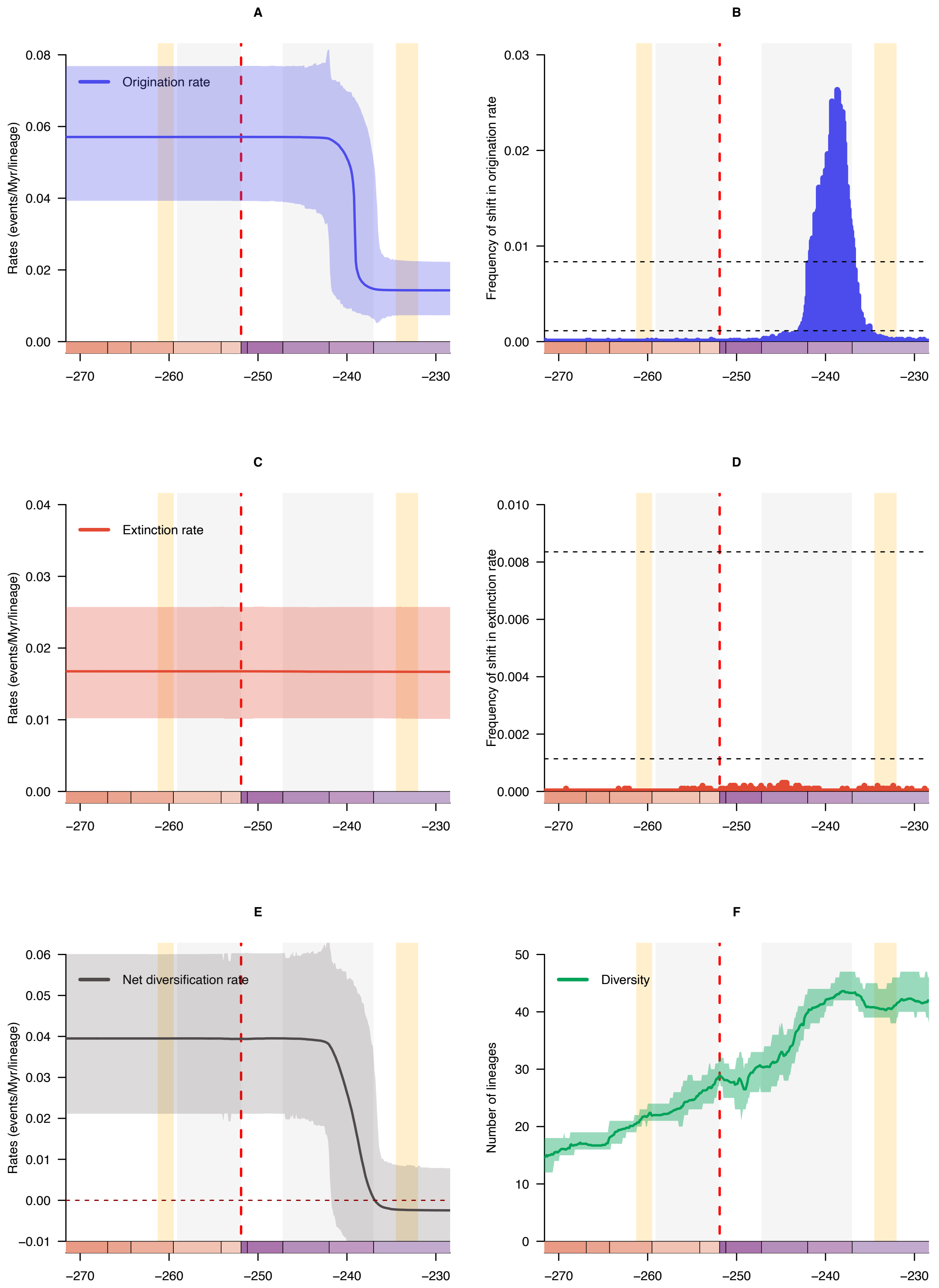

Supplementary Figure 14. Diversification and diversity dynamics of all Palaeoptera families. Bayesian estimations of origination and extinction rates through time as inferred by PyRate using reversible jump Markov Chain Monte Carlo. Marginal estimates of origination rates (A) and extinction rates (C) through time are shown as mean and 95% credibility intervals (shaded areas). The frequency of a sampled rate shift is computed within small time bins for origination and extinction rates (B and D, respectively), with horizontal dashed lines indicating log-Bayes factors of 2 (bottom) and 6 (top). Sampling frequencies higher than log-Bayes factors = 6 indicate strong statistical support for a rate shift. Panel (E) shows net diversification rates through time (computed as the posterior difference between origination and extinction rates through time). Panel (F) shows the number of families through time computed by summing up the lifespans of all genus. For each plot, solid lines indicate mean posterior rates; shaded areas show 95% CI. The red vertical line indicates the Permian-Triassic boundary. First orange period represents the GEE, the second is the CPE. Time is in millions of years. The color of each period in the chronostratigraphic scale follows that of the International Chronostratigraphic Chart (v2022/02).

Supplementary Figure 14.

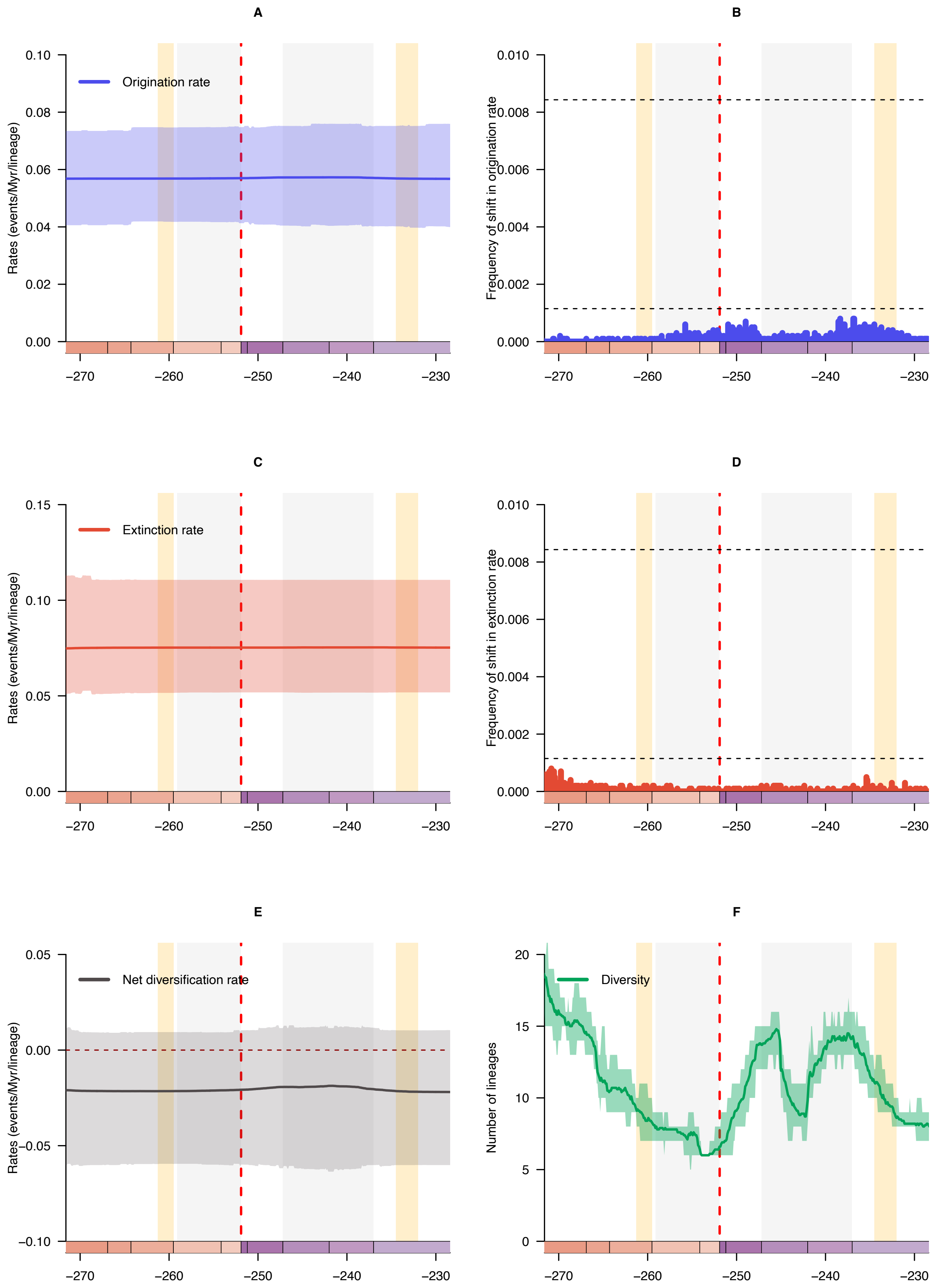

Supplementary Figure 15. Diversification and diversity dynamics of all Polyneoptera families. Bayesian estimations of origination and extinction rates through time as inferred by PyRate using reversible jump Markov Chain Monte Carlo. Marginal estimates of origination rates (A) and extinction rates (C) through time are shown as mean and 95% credibility intervals (shaded areas). The frequency of a sampled rate shift is computed within small time bins for origination and extinction rates (B and D, respectively), with horizontal dashed lines indicating log-Bayes factors of 2 (bottom) and 6 (top). Sampling frequencies higher than log-Bayes factors = 6 indicate strong statistical support for a rate shift. Panel (E) shows net diversification rates through time (computed as the posterior difference between origination and extinction rates through time). Panel (F) shows the number of families through time computed by summing up the lifespans of all genus. For each plot, solid lines indicate mean posterior rates; shaded areas show 95% CI. The red vertical line indicates the Permian-Triassic boundary. First orange period represents the GEE, the second is the CPE. Time is in millions of years. The color of each period in the chronostratigraphic scale follows that of the International Chronostratigraphic Chart (v2022/02).

Supplementary Figure 15.

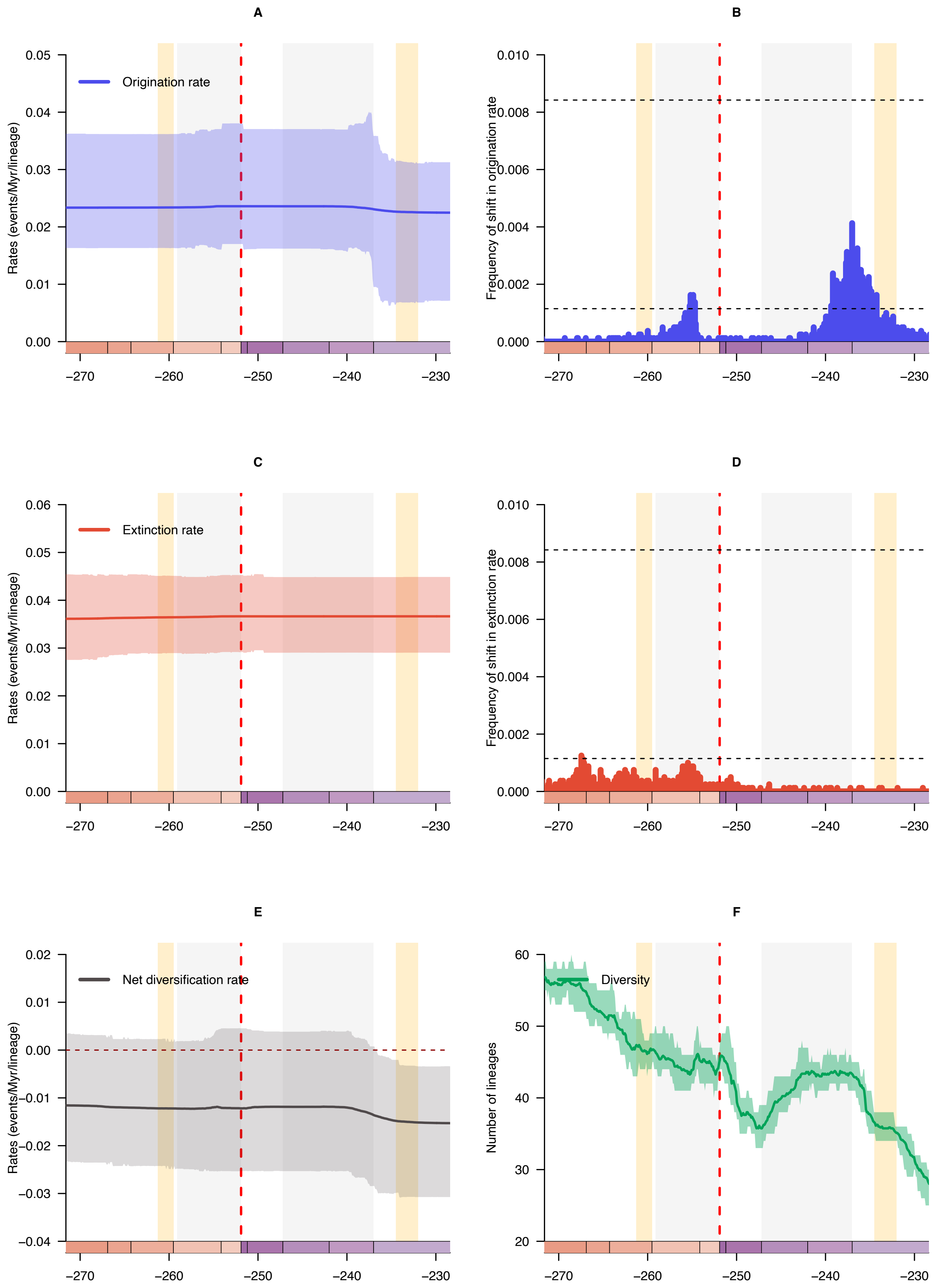

Supplementary Figure 16. Diversification and diversity dynamics of all insect families without singleton. Bayesian estimations of origination and extinction rates through time as inferred by PyRate using reversible jump Markov Chain Monte Carlo. Marginal estimates of origination rates (A) and extinction rates (C) through time are shown as mean and 95% credibility intervals (shaded areas). The frequency of a sampled rate shift is computed within small time bins for origination and extinction rates (B and D, respectively), with horizontal dashed lines indicating log-Bayes factors of 2 (bottom) and 6 (top). Sampling frequencies higher than log-Bayes factors = 6 indicate strong statistical support for a rate shift. Panel (E) shows net diversification rates through time (computed as the posterior difference between origination and extinction rates through time). Panel (F) shows the number of families through time computed by summing up the lifespans of all genus. For each plot, solid lines indicate mean posterior rates; shaded areas show 95% CI. The red vertical line indicates the Permian-Triassic boundary. First orange period represents the GEE, the second is the CPE. Time is in millions of years. The color of each period in the chronostratigraphic scale follows that of the International Chronostratigraphic Chart (v2022/02).

Supplementary Figure 16.

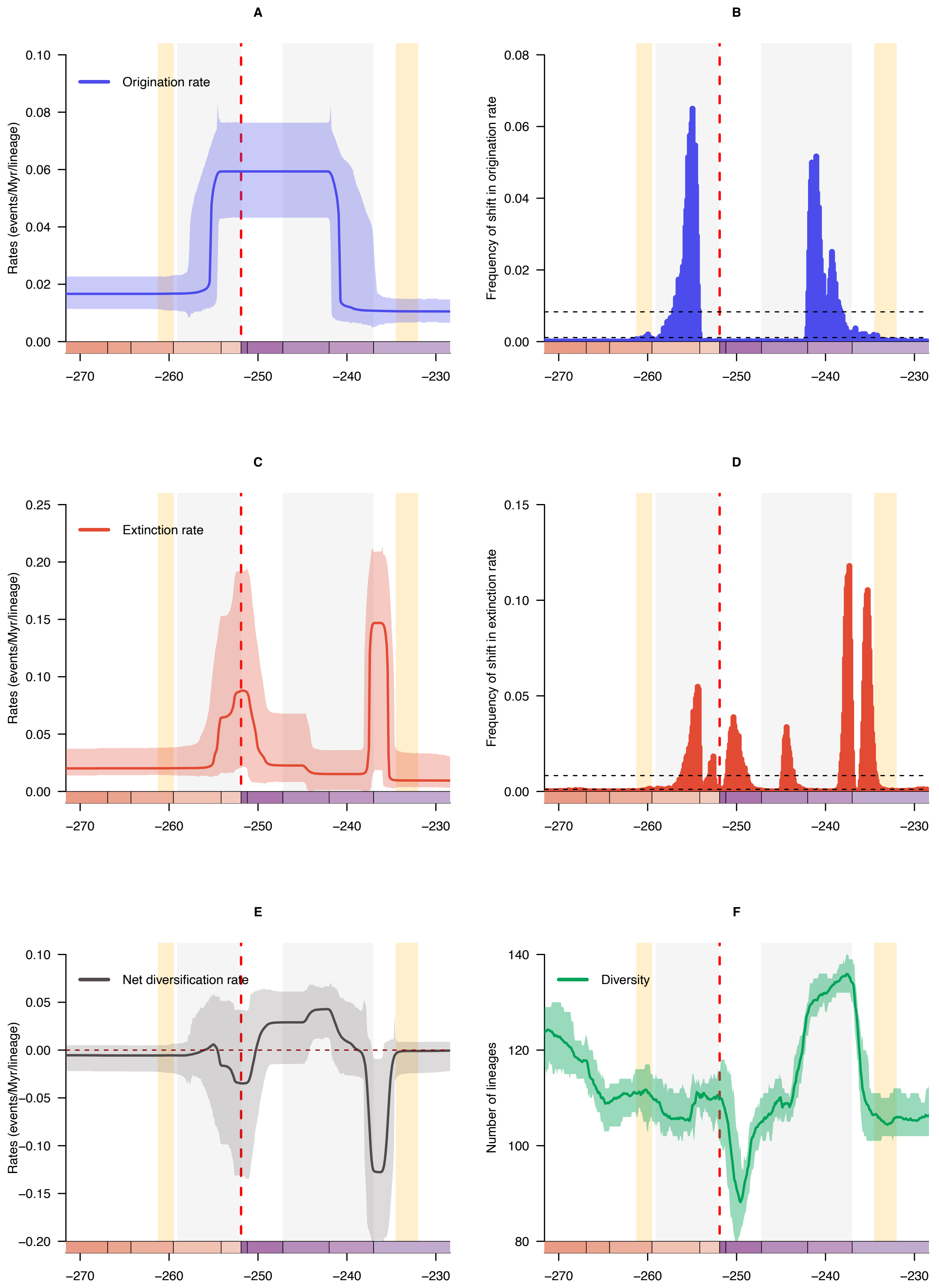

Supplementary Figure 17. Estimate of the mean preservation rates (i.e. number of occurrences per species per Myr) and the variations though time inferred from PyRate. Solid lines indicate mean posterior rates and shaded areas show 95% CI. The red vertical line indicates the Permian-Triassic boundary. First orange period represents the GEE, the second is the CPE. Time is in millions of years. The color of each period in the chronostratigraphic scale follows that of the International Chronostratigraphic Chart (v2022/02).

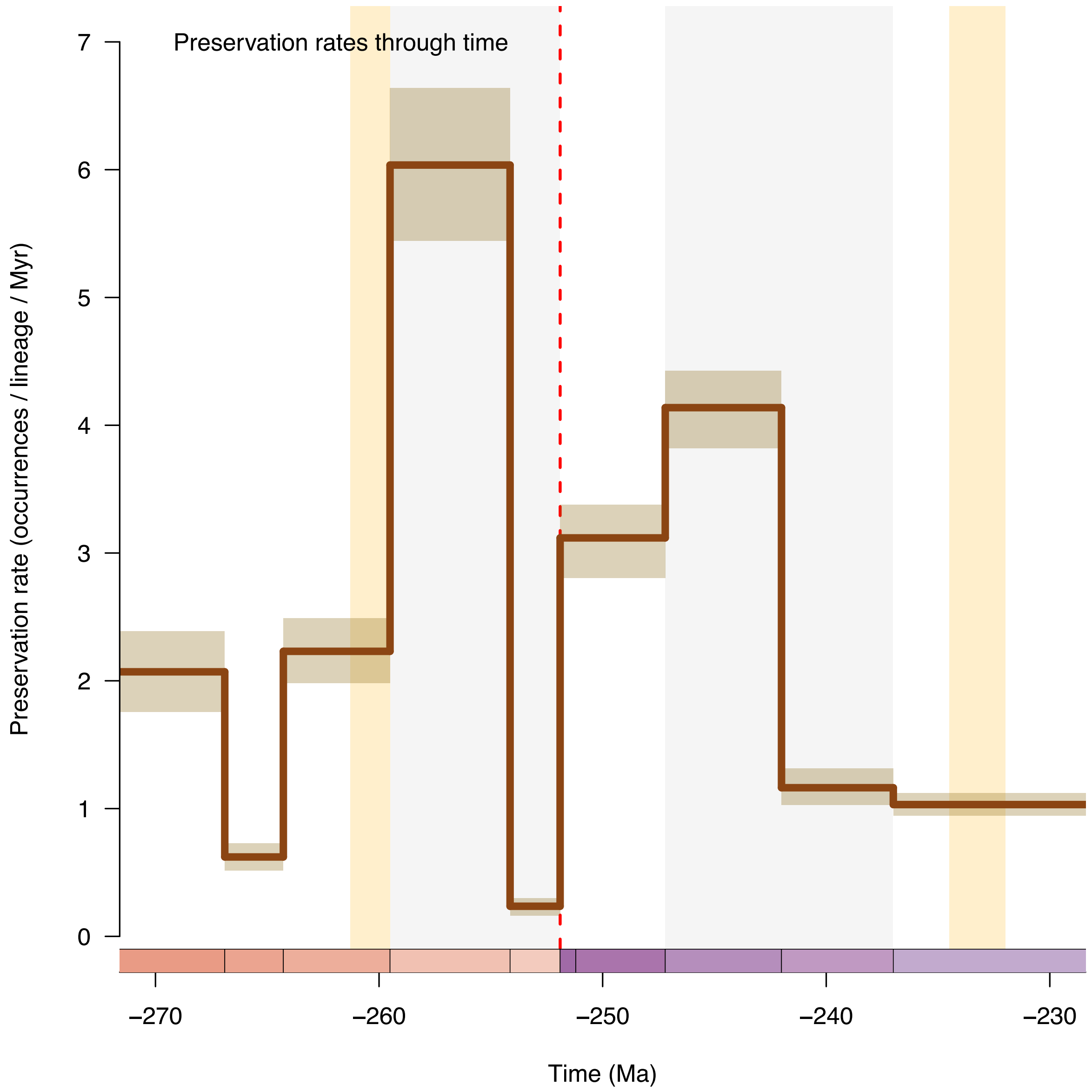

Supplementary Figure 18. Dynamics of insects during Permo–Triassic, incorporating effect of putative factors. A Dynamics of origination and extinction rates through time as estimated with Bayesian multivariate birth–death model in PyRate. B Dynamic of net diversification rate through time. Solid lines mean posterior rates, shaded areas 95% CI. The red vertical line indicates the Permian-Triassic boundary. First orange period represents the GEE, the second is the CPE. Time is in millions of years. The color of each period in the chronostratigraphic scale follows that of the International Chronostratigraphic Chart (v2022/02).

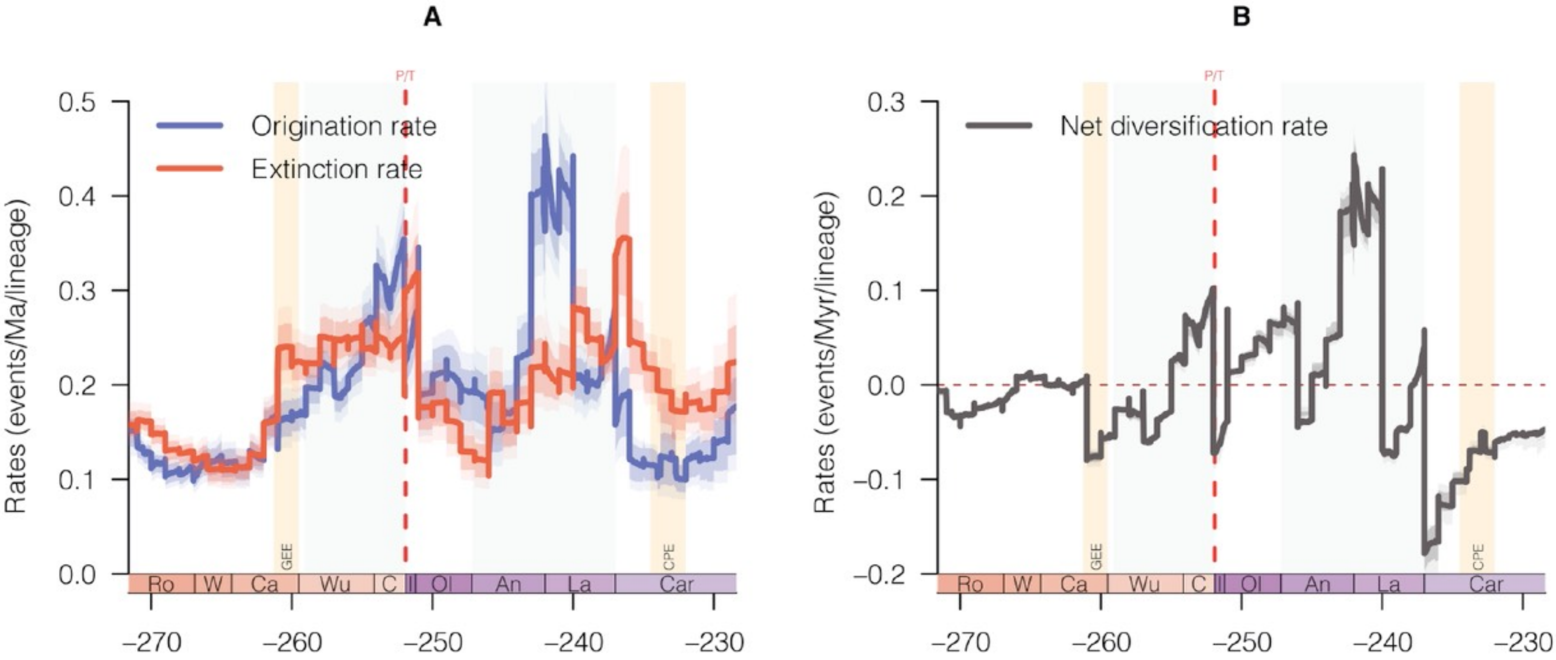

Supplementary Figure 19. Diversification and diversity dynamics of generalist insects. Bayesian estimations of origination and extinction rates through time as inferred by PyRate using reversible jump Markov Chain Monte Carlo. Marginal estimates of origination rates (A) and extinction rates (C) through time are shown as mean and 95% credibility intervals (shaded areas). The frequency of a sampled rate shift is computed within small time bins for origination and extinction rates (B and D, respectively), with horizontal dashed lines indicating log-Bayes factors of 2 (bottom) and 6 (top). Sampling frequencies higher than log-Bayes factors = 6 indicate strong statistical support for a rate shift. Panel (E) shows net diversification rates through time (computed as the posterior difference between origination and extinction rates through time). For each plot, solid lines indicate mean posterior rates; shaded areas show 95% CI. The red vertical line indicates the Permian-Triassic boundary. First orange period represents the GEE, the second is the CPE. Time is in millions of years. The color of each period in the chronostratigraphic scale follows that of the International Chronostratigraphic Chart (v2022/02).

Supplementary Figure 19.

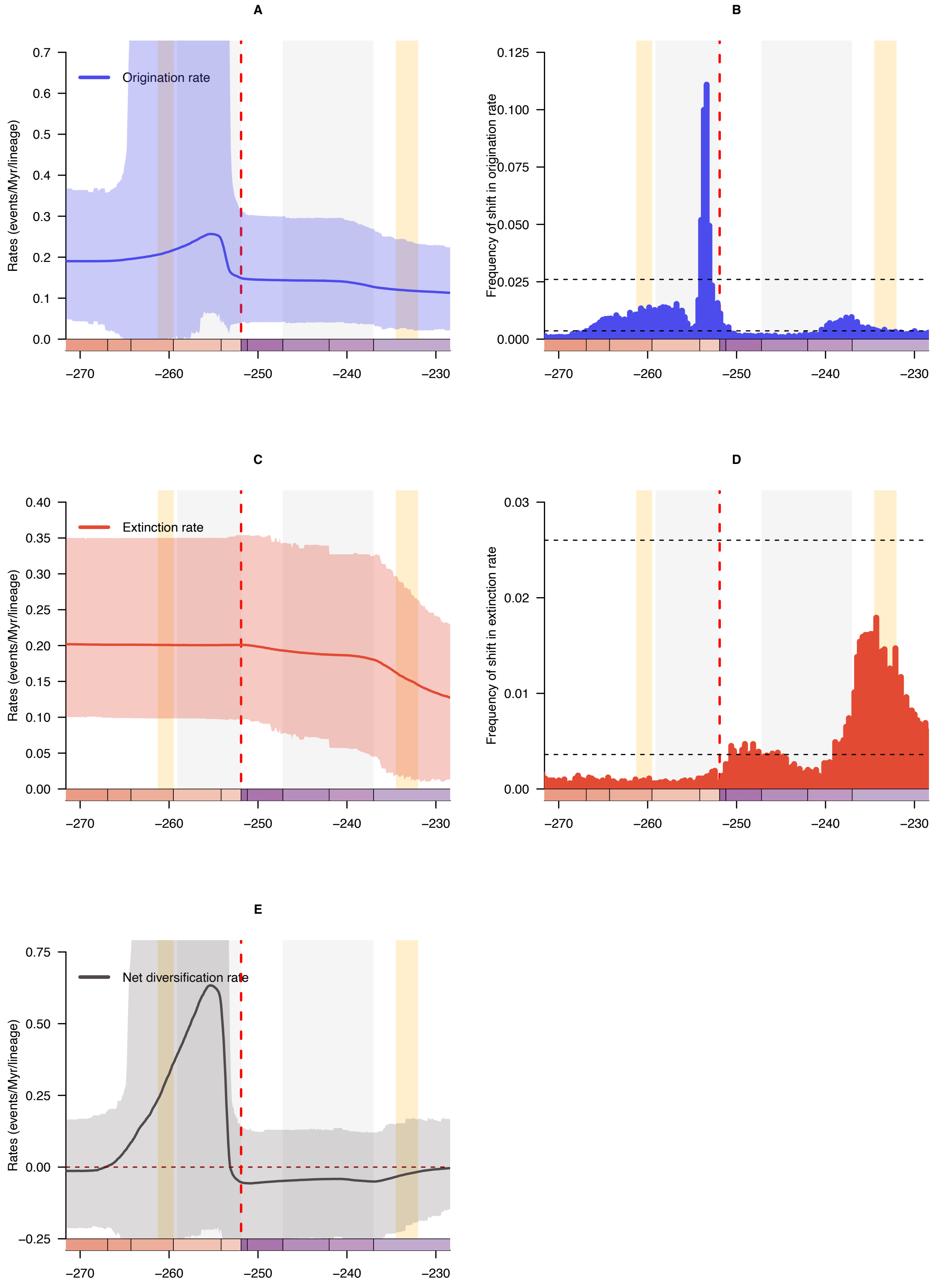

Supplementary Figure 20. Diversification and diversity dynamics of detritivores/fungivores. Bayesian estimations of origination and extinction rates through time as inferred by PyRate using reversible jump Markov Chain Monte Carlo. Marginal estimates of origination rates (A) and extinction rates (C) through time are shown as mean and 95% credibility intervals (shaded areas). The frequency of a sampled rate shift is computed within small time bins for origination and extinction rates (B and D, respectively), with horizontal dashed lines indicating log-Bayes factors of 2 (bottom) and 6 (top). Sampling frequencies higher than log-Bayes factors = 6 indicate strong statistical support for a rate shift. Panel (E) shows net diversification rates through time (computed as the posterior difference between origination and extinction rates through time). For each plot, solid lines indicate mean posterior rates; shaded areas show 95% CI. The red vertical line indicates the Permian-Triassic boundary. First orange period represents the GEE, the second is the CPE. Time is in millions of years. The color of each period in the chronostratigraphic scale follows that of the International Chronostratigraphic Chart (v2022/02).

Supplementary Figure 20.

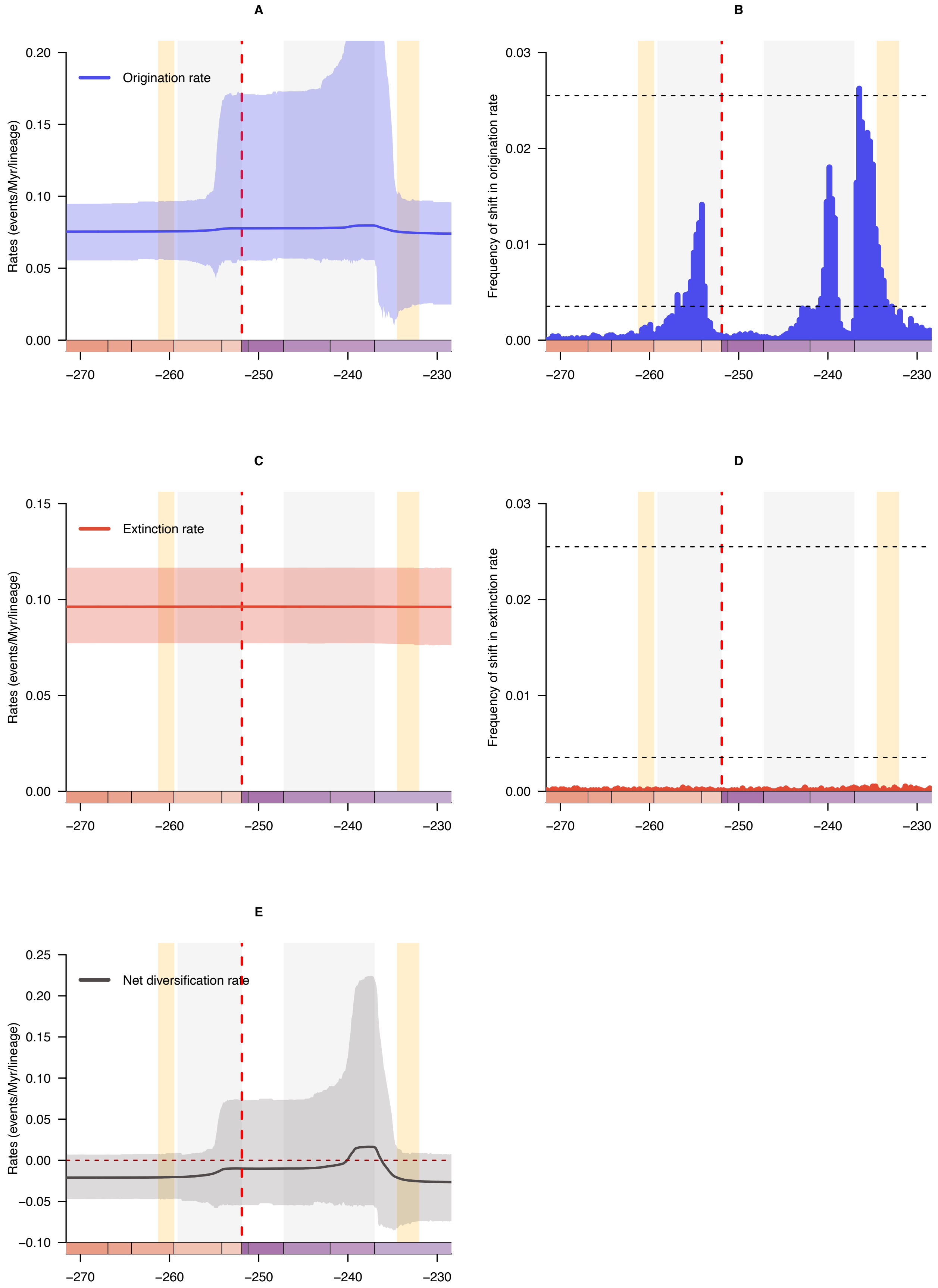

Supplementary Figure 21. Diversification and diversity dynamics of phytophagous insects. Bayesian estimations of origination and extinction rates through time as inferred by PyRate using reversible jump Markov Chain Monte Carlo. Marginal estimates of origination rates (A) and extinction rates (C) through time are shown as mean and 95% credibility intervals (shaded areas). The frequency of a sampled rate shift is computed within small time bins for origination and extinction rates (B and D, respectively), with horizontal dashed lines indicating log-Bayes factors of 2 (bottom) and 6 (top). Sampling frequencies higher than log-Bayes factors = 6 indicate strong statistical support for a rate shift. Panel (E) shows net diversification rates through time (computed as the posterior difference between origination and extinction rates through time). For each plot, solid lines indicate mean posterior rates; shaded areas show 95% CI. The red vertical line indicates the Permian-Triassic boundary. First orange period represents the GEE, the second is the CPE. Time is in millions of years. The color of each period in the chronostratigraphic scale follows that of the International Chronostratigraphic Chart (v2022/02).

Supplementary Figure 21.

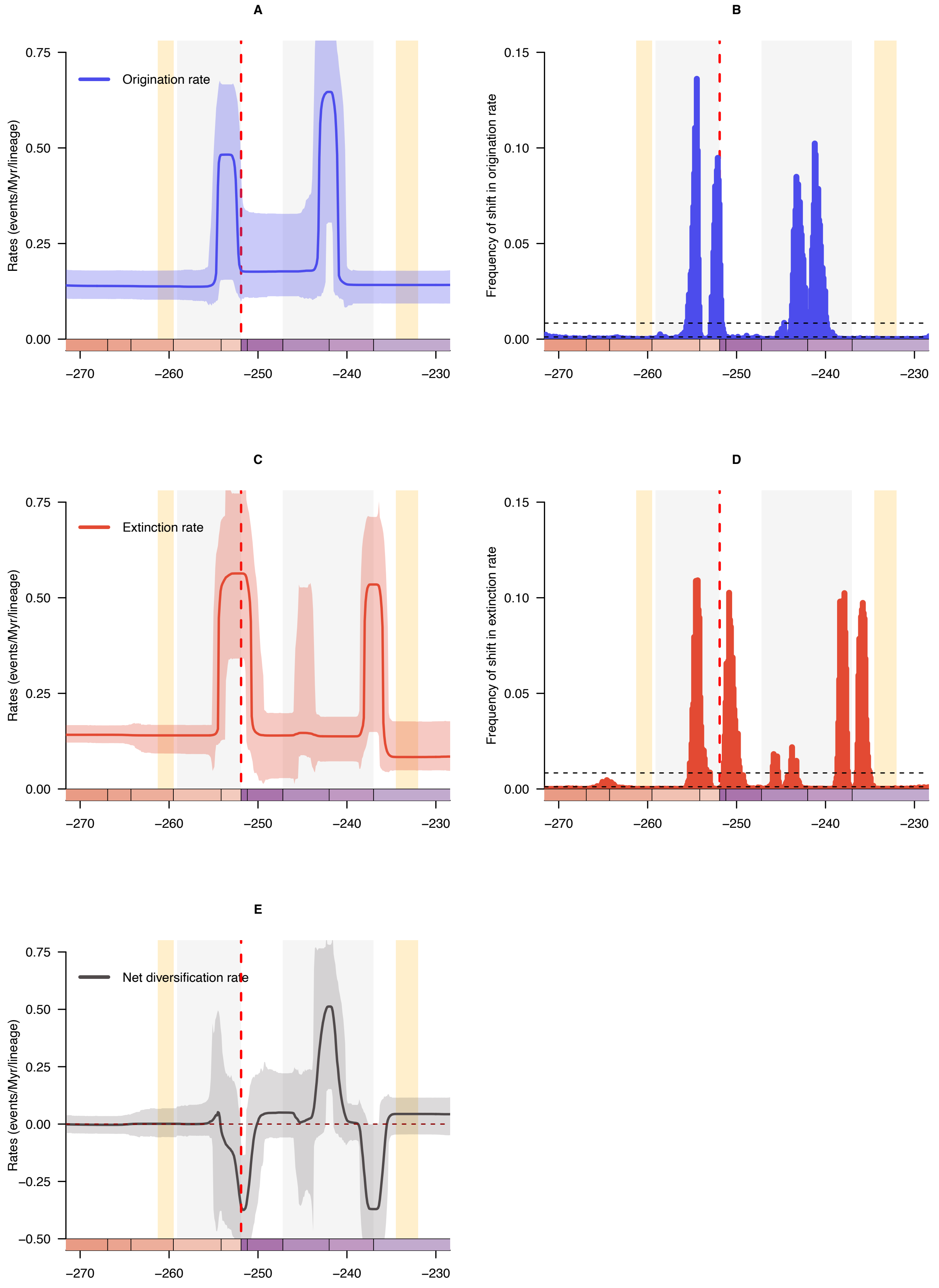

Supplementary Figure 22. Diversification and diversity dynamics of predators. Bayesian estimations of origination and extinction rates through time as inferred by PyRate using reversible jump Markov Chain Monte Carlo. Marginal estimates of origination rates (A) and extinction rates (C) through time are shown as mean and 95% credibility intervals (shaded areas). The frequency of a sampled rate shift is computed within small time bins for origination and extinction rates (B and D, respectively), with horizontal dashed lines indicating log-Bayes factors of 2 (bottom) and 6 (top). Sampling frequencies higher than log-Bayes factors = 6 indicate strong statistical support for a rate shift. Panel (E) shows net diversification rates through time (computed as the posterior difference between origination and extinction rates through time). For each plot, solid lines indicate mean posterior rates; shaded areas show 95% CI. The red vertical line indicates the Permian-Triassic boundary. First orange period represents the GEE, the second is the CPE. Time is in millions of years. The color of each period in the chronostratigraphic scale follows that of the International Chronostratigraphic Chart (v2022/02).

Supplementary Figure 22.

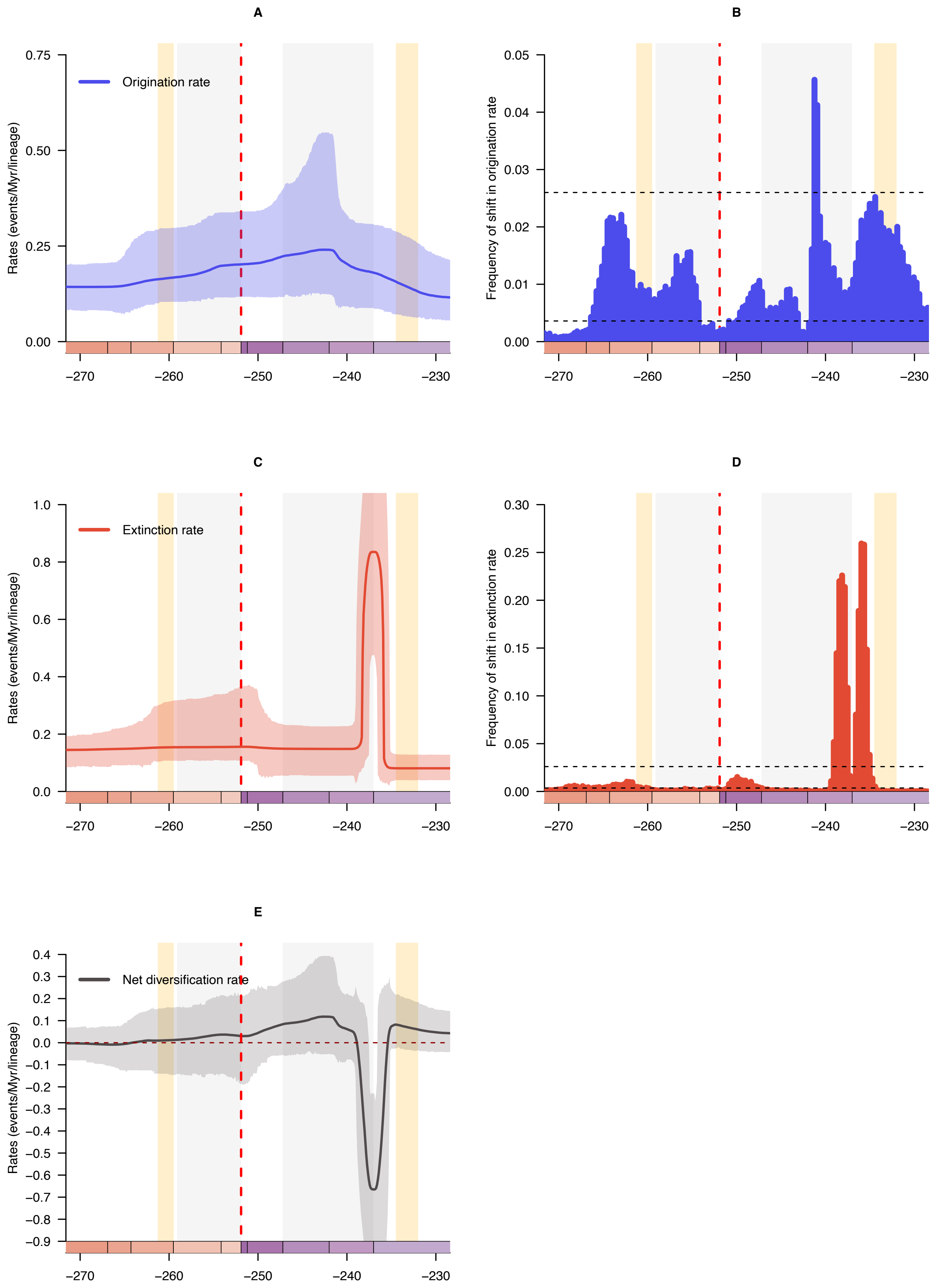

Supplementary Figure 23. Diversification and diversity dynamics of non-herbivores and non-predators. Bayesian estimations of origination and extinction rates through time as inferred by PyRate using reversible jump Markov Chain Monte Carlo. Marginal estimates of origination rates (A) and extinction rates (C) through time are shown as mean and 95% credibility intervals (shaded areas). The frequency of a sampled rate shift is computed within small time bins for origination and extinction rates (B and D, respectively), with horizontal dashed lines indicating log-Bayes factors of 2 (bottom) and 6 (top). Sampling frequencies higher than log-Bayes factors = 6 indicate strong statistical support for a rate shift. Panel (E) shows net diversification rates through time (computed as the posterior difference between origination and extinction rates through time). For each plot, solid lines indicate mean posterior rates; shaded areas show 95% CI. The red vertical line indicates the Permian-Triassic boundary. First orange period represents the GEE, the second is the CPE. Time is in millions of years. The color of each period in the chronostratigraphic scale follows that of the International Chronostratigraphic Chart (v2022/02).

Supplementary Figure 23.

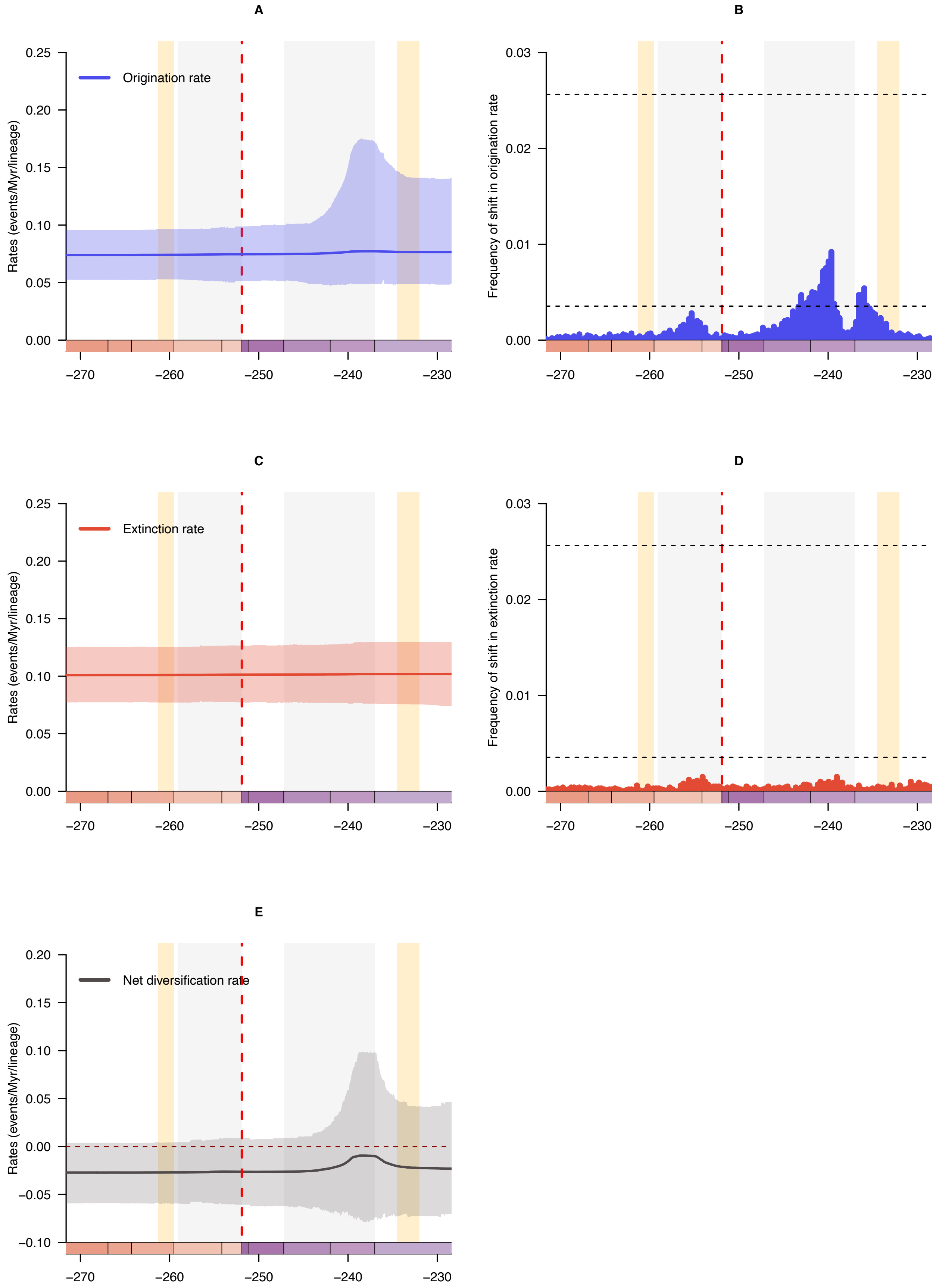

Supplementary Figure 24. Diversity trajectories and the effect of diversity dependence or facilitation for guilds of insects. A Diversity trajectories of four guilds of insects between Roadian and Carnian. Reconstructions of diversity trajectories replicated 10 times, incorporating uncertainties around ages of the fossil occurrences. For each plot, solid lines indicate mean posterior rates; shaded areas show 95% CI. First orange period represents the GEE, the second is the CPE. Time is in millions of years. The color of each period in the chronostratigraphic scale follows that of the International Chronostratigraphic Chart (v2022/02). **B** Network showing positive and negative interactions within and between guilds (only significant correlations are shown). Each arrow indicates the intensity of interaction imposed by a given guilds toward another one. Silhouettes from http://phylopic.org/. Titanoptera (by Melissa Broussard), and Trichoptera (by Didier Descouens; vectorized by T. Michael Keesey) licenses at https://creativecommons.org/licenses/by-sa/3.0/; Coleoptera, Dictyoptera, Hemiptera, Hymenoptera, Libellulidae, Mecoptera, Miomoptera, Palaeodictyoptera, Plecoptera, and Psocodea licenses at https://creativecommons.org/publicdomain/zero/1.0/; Dermaptera, Thysanoptera, and Coleoptera by Corentin Jouault.

Supplementary Figure 24.

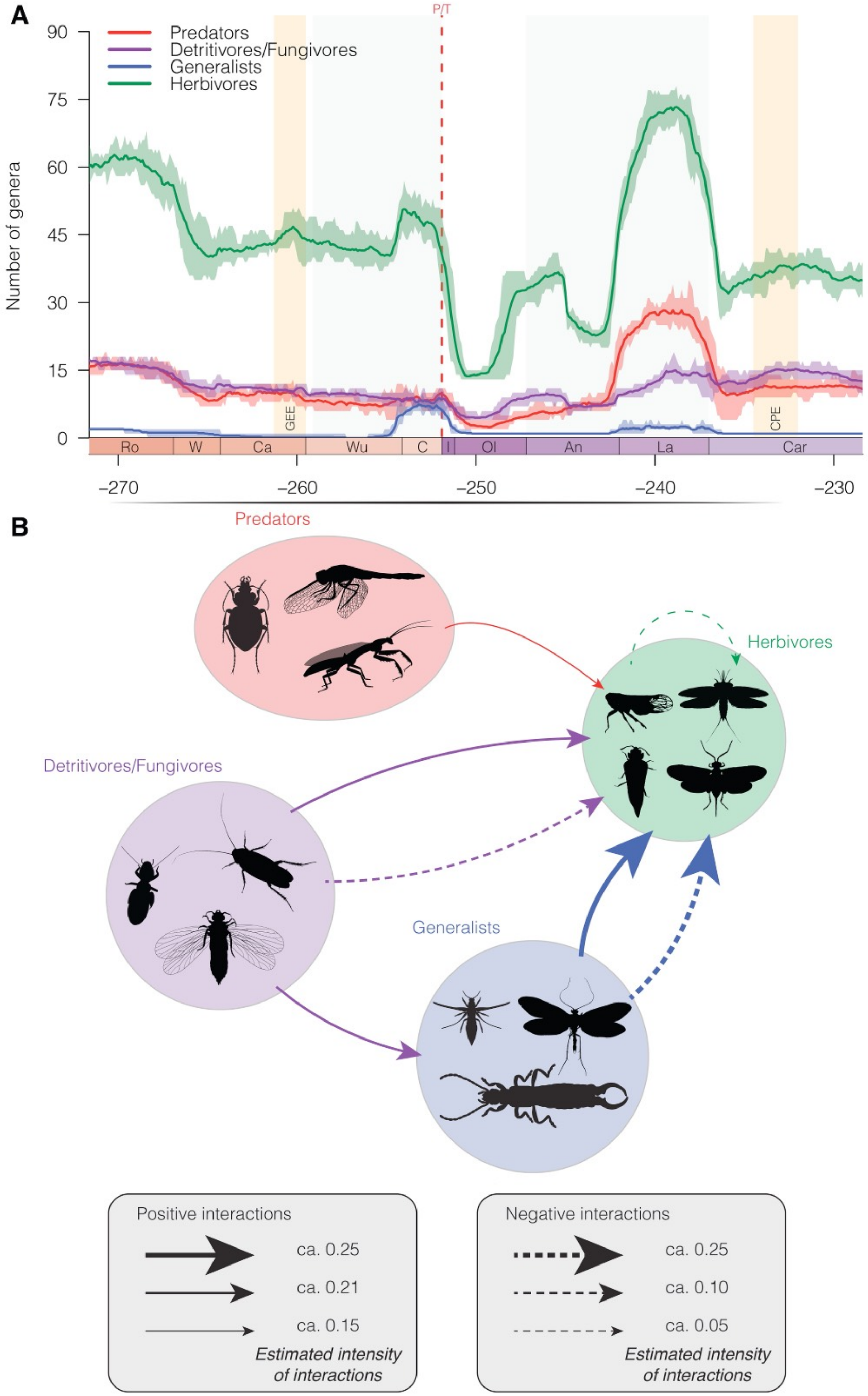

	Severity of each event				
	**Survivor**	**Extinct genera**	**Percentage survivor**	**Percentage extinction**	**Total of genera**
R/W	105	191	35.47	64.53	296
LPME	45	214	17.37	82.63	259
L/C	94	280	25.13	74.87	374

Supplementary Table 1. Severity of each event with estimate of survivors and extinction percentage.

Predecline					
**Parameters**	**q**	**alpha**	**Weibull shape**	**Weibull scale**	**Mean longevity (Myrs)**
mean	0.6623	0.1791	2.4824	4.505	4.127
median	0.6535	0.171	2.258	4.6	4.176
95% HPD	[0.4522, 0.8947]	[0.121, 0.258]	[0.61, 4.725]	[2.263, 6,4917]	[2.349, 5.84]

Decline					
**Parameters**	**q**	**alpha**	**Weibull shape**	**Weibull scale**	**Mean longevity (Myrs)**
Mean	2.0093	0.2517	**9.1677**	2.1879	2.0571
Median	2.0119	0.2469	**8.0317**	2.2001	2.0755
95% HDP	[1.4673, 2.5036]	[0.1934, 0.3159]	**[2.3886, 18.7343]**	[1.9598, 2.3936]	[1.785, 2.3047]

Post-crisis					
**Parameters**	**q**	**alpha**	**Weibull shape**	**Weibull scale**	**Mean longevity**
Mean	0.131555556	0.831655556	1.884166667	10.44302	9.060744444
Median	0.128844444	0.617511111	1.766144444	10.0966	9.1425
95% HDP	[0.0866, 0.1811]	[0.2342, 2.2485]	[0.7421, 3.2443]	[4.7918, 14.2935]	[4.9712, 12.75679]

Supplementary Table 2. Posterior parameter estimates for the age-dependent extinction (ADE) model for insects genera. Preservation rates (q) are estimated for the predecline, the decline and the post-crisis separately. Bold values for the shape parameter of the Weibull distribution indicate a significant effect of taxon age on extinction rates. A Weibull shape significantly greater than 1 indicates that extinction probability increases with species age, while a Weibull shape significantly lesser than 1 indicates that extinction rate is higher in younger taxa (Weibull shape not significantly different from 1 means no effect of age on extinction rates).

Supplementary Table 3.

Parameters		Median	95% HPD interval
Baseline rates	λ0	0.0724	[4.843E-4, 0.4762]
	μ0	0.2228	[1.3297E-3, 0.8564]
Correlation parameters to origination	*G*λ0_0	-0.6953	[-1.448, 0.1982]
	*G*λ0_1	-0.0001	[-0.0012, 3.9499E-4]
	*G*λ0_2	-1.5249	[-2.9965, 0.12]
	*G*λ0_3	21.0655	[-2.427, 47.9085]
	*G*λ0_4	2.2448	[-0.6884, 6.4625]
	***G*λ0_5**	**-30.8302**	**[-44.8738, -19.5187]**
	*G*λ0_6	0.1989	[-6.4519, 14.3636]
	*G*λ0_7	-0.0264	[-0.1522, 0.0382]
	***G*λ0_8**	**3.4209**	**[0.9734, 5.1021]**
Correlation parameters to extinction	*G*μ0_0	-0.1697	[-0.9393, 0.3406]
	*G*μ0_1	2.3589E-6	[-0.0004, 4.3525E-4]
	*G*μ0_2	-0.3251	[-1.4964, 0.5619]
	*G*μ0_3	-21.8994	[-48.574, 2.2569]
	***G*μ0_4**	**-5.0208**	**[-8.504, -1.5169]**
	*G*μ0_5	12.3112	[-0.9476, 24.4872]
	*G*μ0_6	0.3314	[-4.6098, 11.9277]
	*G*μ0_7	-0.0009	[-0.0753, 0.0675]
	***G*μ0_8**	**2.7087**	**[1.1493, 4.4888]**
Shrinkage weights (origination)	ωλ0_0	0.6096	[0.0493, 1]
	ωλ0_1	0.7437	[0.018, 1]
	ωλ0_2	0.8024	[0.167, 1]
	ωλ0_3	0.9548	[0.1633, 1]
	ωλ0_4	0.7965	[0.04, 1]
	ωλ0_5	0.9986	[0.9926, 1]
	ωλ0_6	0.8191	[0.0223, 1]
	ωλ0_7	0.7948	[0.0257, 1]
	ωλ0_8	0.9253	[0.5917, 1]
Shrinkage weights (extinction)	ωμ0_0	0.3492	[3.9668E-9, 0.9599]
	ωμ0_1	0.5743	[1.2691E-8, 0.9794]
	ωμ0_2	0.4827	[1.5274E-9, 0.9735]
	ωμ0_3	0.9592	[0.2317, 1]
	ωμ0_4	0.9219	[0.566, 1]
	ωμ0_5	0.9902	[0.3168, 1]
	ωμ0_6	0.8105	[0.0235, 1]
	ωμ0_7	0.6388	[2.896E-6, 0.9829]
	ωμ0_8	0.9041	[0.5424, 1]
Global shrinkage Hyperprior	τ	2.2791	[0.5951, 4.9989]
	η	4.1462	[2.1027, 6.4146]

Supplementary Table 3. Posterior parameter estimates for the MBD model applied to all insect genera for the Permo-Triassic period. Baseline origination and extinction rates (λ0 and μ0) and correlation parameters (*G*λ and *G*μ). The drivers are numbered as follows: (0) diversity of all insects through time, (1) global variation of atmospheric CO_2 through time, (2) global variation of atmospheric O_2 through time, (3) continental fragmentation through time, (4) gymnosperm diversity through time, (5) Polypodiales ferns diversity through time, (6) Spore-plants diversity through time, (7) global temperature changes through time, and (8) non-Polypodiales ferns diversity through time. Shrinkage weights (ω), based on local and global shrinkage parameters, and global shrinkage (τ). Shrinkage weights greater than 0.5 (highlighted in bold) indicate significant evidence for correlation (positive or negative depending on the respective *G*λ or *G*μ value).

Supplementary Table 4.

Parameters		Median	95% HPD interval
Baseline rates	λ0	0.3335	[0.0112, 1.1506]
	μ0	0.4882	[0.014, 1.451]
Correlation parameters to origination	*G*λ0_0	-0.0259	[-2.1271, 1.8338]
	*G*λ0_1	0	[-0.0019, 1.6662E-3]
	*G*λ0_2	-0.0519	[-3.7964, 2.8628]
	*G*λ0_3	-0.082	[-44.3637, 29.622]
	*G*λ0_4	-0.0162	[-6.7293, 6.1707]
	*G*λ0_5	-0.0018	[-9.4838, 10.9702]
	*G*λ0_6	-0.0074	[-7.4152, 6.8256]
	*G*λ0_7	-0.0012	[-0.0849, 0.0637]
	*G*λ0_8	-0.127	[-4.6372, 2.4989]
Correlation parameters to extinction	*G*μ0_0	-0.2502	[-3.0874, 1.5379]
	*G*μ0_1	2.3102E-6	[-0.0022, 2.0958E-3]
	*G*μ0_2	0.1166	[-5.6091, 17.1023]
	*G*μ0_3	-0.8361	[-563.8765, 35.7958]
	*G*μ0_4	0.57	[-4.8817, 22.319]
	*G*μ0_5	9.7442E-5	[-9.4709, 9.6931]
	*G*μ0_6	6.6707E-3	[-19.6512, 17.8137]
	*G*μ0_7	2.4926E-3	[-0.0985, 0.4781]
	*G*μ0_8	-1.0055	[-18.6991, 2.7]
Shrinkage weights (origination)	ωλ0_0	0.2703	[2.1664E-10, 0.9508]
	ωλ0_1	0.4105	[6.2446E-10, 0.9941]
	ωλ0_2	0.314	[4.7266E-9, 0.9729]
	ωλ0_3	0.4347	[7.8629E-9, 0.9934]
	ωλ0_4	0.3585	[9.5895E-10, 0.9782]
	ωλ0_5	0.4295	[4.3018E-9, 0.9945]
	ωλ0_6	0.3944	[7.7646E-8, 0.9899]
	ωλ0_7	0.3167	[9.4246E-8, 0.9738]
	ωλ0_8	0.3795	[3.5417E-8, 0.9777]
Shrinkage weights (extinction)	ωμ0_0	0.4558	[7.19E-12, 0.9697]
	ωμ0_1	0.4318	[3.1242E-9, 0.996]
	ωμ0_2	0.473	[1.1604E-9, 0.9964]
	ωμ0_3	0.6041	[2.2565E-3, 1]
	ωμ0_4	0.6237	[2.4073E-9, 0.9957]
	ωμ0_5	0.4315	[9.5733E-8, 0.9938]
	ωμ0_6	0.4643	[1.9377E-3, 1]
	ωμ0_7	0.4936	[8.461E-9, 0.996]
	ωμ0_8	0.752	[2.5874E-3, 1]
Global shrinkage Hyperprior	τ	0.8985	[2.8272E-3, 3.3166]
	η	3.9197	[2.1333, 6.5276]

Supplementary Table 4. Posterior parameter estimates for the MBD model applied to all insect genera for the Roadian-Wordian boundary. Baseline origination and extinction rates (λ0 and μ0) and correlation parameters (*G*λ and *G*μ). The drivers are numbered as follows: (0) diversity of all insects through time, (1) global variation of atmospheric CO_2 through time, (2) global variation of atmospheric O_2 through time, (3) continental fragmentation through time, (4) gymnosperm diversity through time, (5) Polypodiales ferns diversity through time, (6) Spore-plants diversity through time, (7) global temperature changes through time, and (8) non-Polypodiales ferns diversity through time. Shrinkage weights (ω), based on local and global shrinkage parameters, and global shrinkage (τ). Shrinkage weights greater than 0.5 (highlighted in bold) indicate significant evidence for correlation (positive or negative depending on the respective *G*λ or *G*μ value).

Supplementary Table 5.

Parameters		Median	95% HPD Interval
Baseline rates	λ0	0.3484	[9.1542E-4, 1.2764]
	μ0	0.4598	[0.0116, 1.4412]
Correlation parameters to origination	*G*λ0_0	2.0857	[-1.4726, 8.2998]
	*G*λ0_1	0	[-0.0088, 4.8625E-3]
	*G*λ0_2	3.9582	[-1.2356, 12.0288]
	*G*λ0_3	-19.4186	[-395.2552, 37.8694]
	*G*λ0_4	2.2649	[-4.5384, 15.1251]
	*G*λ0_5	-0.0013	[-21.6867, 25.8842]
	*G*λ0_6	-1.4504	[-51.9712, 7.1806]
	*G*λ0_7	-0.0329	[-0.364, 0.1141]
	*G*λ0_8	0.2455	[-4.4952, 8.8946]
Correlation parameters to extinction	*G*μ0_0	-1.1768	[-4.3181, 0.8558]
	*G*μ0_1	8.9765E-7	[-0.004, 4.5939E-3]
	*G*μ0_2	-0.2889	[-6.6422, 3.3022]
	*G*μ0_3	-1.6232	[-145.2185, 45.3098]
	*G*μ0_4	-7.1985	[-22.7294, 0.9237]
	*G*μ0_5	4.4297E-4	[-24.7322, 23.41]
	*G*μ0_6	0.0761	[-22.7127, 24.069]
	*G*μ0_7	3.2867E-5	[-0.1787, 0.1154]
	*G*μ0_8	3.8683	[-1.2655, 15.7564]
Shrinkage weights (origination)	ωλ0_0	0.8949	[0.0496, 1]
	ωλ0_1	0.8719	[0.0271, 1]
	ωλ0_2	0.9469	[0.0693, 1]
	ωλ0_3	0.9831	[0.0831, 1]
	ωλ0_4	0.885	[0.0353, 1]
	ωλ0_5	0.8602	[0.0215, 1]
	ωλ0_6	0.9442	[0.0395, 1]
	ωλ0_7	0.9119	[0.0269, 1]
	ωλ0_8	0.7933	[0.0181, 1]
Shrinkage weights (extinction)	ωμ0_0	0.793	[0.019, 1]
	ωμ0_1	0.8559	[0.0186, 1]
	ωμ0_2	0.758	[0.0151, 1]
	ωμ0_3	0.8706	[0.0235, 1]
	ωμ0_4	0.9604	[0.14, 1]
	ωμ0_5	0.8493	[0.023, 1]
	ωμ0_6	0.8556	[0.0192, 1]
	ωμ0_7	0.7524	[0.0125, 1]
	ωμ0_8	0.9504	[0.0382, 1]
Global shrinkage Hyperprior	τ	2.5804	[0.2064, 7.2422]
	η	3.8485	[1.7704, 7.7229]

Supplementary Table 5. Posterior parameter estimates for the MBD model applied to all insect genera for the LPME. Baseline origination and extinction rates (λ0 and μ0) and correlation parameters (*G*λ and *G*μ). The drivers are numbered as follows: (0) diversity of all insects through time, (1) global variation of atmospheric CO_2 through time, (2) global variation of atmospheric O_2 through time, (3) continental fragmentation through time, (4) gymnosperm diversity through time, (5) Polypodiales ferns diversity through time, (6) Spore-plants diversity through time, (7) global temperature changes through time, and (8) non-Polypodiales ferns diversity through time. Shrinkage weights (ω), based on local and global shrinkage parameters, and global shrinkage (τ). Shrinkage weights greater than 0.5 (highlighted in bold) indicate significant evidence for correlation (positive or negative depending on the respective *G*λ or *G*μ value).

Supplementary Table 6.

Parameters		Median	95% HPD Interval
Baseline rates	λ0	0.3835	[0.0115, 1.3199]
	μ0	0.4198	[4.6705E-3, 1.4094]
Correlation parameters to origination	*G*λ0_0	0.1164	[-1.0453, 2.0384]
	*G*λ0_1	0	[-0.0041, 2.9433E-3]
	*G*λ0_2	-0.2081	[-15.1903, 9.6222]
	*G*λ0_3	-0.2299	[-97.4472, 69.4701]
	*G*λ0_4	0.015	[-9.8994, 23.5632]
	*G*λ0_5	-0.2619	[-20.2213, 10.116]
	*G*λ0_6	-0.0184	[-14.9275, 15.5655]
	*G*λ0_7	-0.0081	[-0.3881, 0.1362]
	*G*λ0_8	0.1895	[-3.0719, 6.0732]
Correlation parameters to extinction	*G*μ0_0	0.3381	[-0.6272, 1.8639]
	*G*μ0_1	-0.0001	[-0.0556, 4.0491E-3]
	*G*μ0_2	-0.0059	[-15.8408, 17.0042]
	*G*μ0_3	-0.4754	[-182.8487, 95.3737]
	*G*μ0_4	32.5182	[-3.5544, 60.2627]
	*G*μ0_5	-0.0169	[-11.9377, 11.0321]
	*G*μ0_6	1.6853	[-14.0185, 204.2691]
	*G*μ0_7	-1.09	[-1.7922, 0.1101]
	***G*μ0_8**	**18.1399**	**[11.6038, 25.5317]**
Shrinkage weights (origination)	ωλ0_0	0.4754	[3.0806E-9, 0.9856]
	ωλ0_1	0.8612	[0.022, 1]
	ωλ0_2	0.8378	[0.0206, 1]
	ωλ0_3	0.8535	[0.0213, 1]
	ωλ0_4	0.7608	[0.0141, 1]
	ωλ0_5	0.8667	[0.0231, 1]
	ωλ0_6	0.8388	[0.0188, 1]
	ωλ0_7	0.7736	[0.0149, 1]
	ωλ0_8	0.7336	[0.0138, 1]
Shrinkage weights (extinction)	ωμ0_0	0.5433	[2.2883E-8, 0.9858]
	ωμ0_1	0.9549	[0.0341, 1]
	ωμ0_2	0.8544	[0.0228, 1]
	ωμ0_3	0.8773	[0.024, 1]
	ωμ0_4	0.9963	[0.2873, 1]
	ωμ0_5	0.8309	[0.0225, 1]
	ωμ0_6	0.9683	[0.0387, 1]
	ωμ0_7	0.9991	[0.3395, 1]
	ωμ0_8	0.9962	[0.9808, 1]
Global shrinkage Hyperprior	τ	2.601	[0.242, 9.7913]
	η	3.548	[1.96, 7.4076]

Supplementary Table 6. Posterior parameter estimates for the MBD model applied to all insect genera for the Ladinian-Carnian boundary. Baseline origination and extinction rates ($\lambda 0$ and $\mu 0$) and correlation parameters ($G\lambda$ and $G\mu$). The drivers are numbered as follows: (0) diversity of all insects through time, (1) global variation of atmospheric CO_2 through time, (2) global variation of atmospheric O_2 through time, (3) continental fragmentation through time, (4) gymnosperm diversity through time, (5) Polypodiales ferns diversity through time, (6) Spore-plants diversity through time, (7) global temperature changes through time, and (8) non-Polypodiales ferns diversity through time. Shrinkage weights (ω), based on local and global shrinkage parameters, and global shrinkage (τ). Shrinkage weights greater than 0.5 (highlighted in bold) indicate significant evidence for correlation (positive or negative depending on the respective $G\lambda$ or $G\mu$ value).

Supplementary Table 7.

Parameters		Median	95% HPD Interval
Baseline rates	λ0	0.2851	[1.6465E-3, 0.98]
	μ0	0.6057	[0.0482, 1.7087]
Correlation parameters to origination	*G*λ0_0	-0.8277	[-1.9117, 0.0691]
	*G*λ0_1	1.4926E-4	[-0.0001, 4.4735E-4]
	***G*λ0_2**	**-4.2881**	**[-6.7509, -2.0505]**
	*G*λ0_3	27.3863	[-3.6136, 57.1765]
	*G*λ0_4	1.2012	[-1.6289, 5.7963]
	*G*λ0_5	0.6374	[-5.9655, 25.4396]
	*G*λ0_6	1.7154	[-2.3572, 13.0723]
	*G*λ0_7	0.0202	[-0.0348, 0.1122]
	*G*λ0_8	1.0701	[-0.6283, 2.8638]
Correlation parameters to extinction	*G*μ0_0	0.2945	[-0.4393, 1.4964]
	*G*μ0_1	-0.0002	[-0.0005, 4.6283E-5]
	*G*μ0_2	-2.9423	[-5.0375, 0.1304]
	*G*μ0_3	-0.3735	[-38.5253, 25.4842]
	*G*μ0_4	4.017	[-0.3255, 7.9793]
	*G*μ0_5	0.4686	[-5.1889, 17.3398]
	*G*μ0_6	11.5168	[-0.5824, 26.95]
	*G*μ0_7	-0.0031	[-0.1059, 0.0815]
	*G*μ0_8	-2.3617	[-5.2494, 0.4199]
Shrinkage weights (origination)	ωλ0_0	0.6418	[0.0653, 1]
	ωλ0_1	0.6536	[0.0259, 1]
	ωλ0_2	0.947	[0.7364, 1]
	ωλ0_3	0.9699	[0.2261, 1]
	ωλ0_4	0.7063	[0.0192, 0.9999]
	ωλ0_5	0.8573	[0.0227, 1]
	ωλ0_6	0.8957	[0.0338, 1]
	ωλ0_7	0.7305	[0.0174, 1]
	ωλ0_8	0.7237	[0.0336, 1]
Shrinkage weights (extinction)	ωμ0_0	0.4488	[7.3131E-10, 0.9699]
	ωμ0_1	0.7399	[0.0479, 1]
	ωμ0_2	0.9024	[0.2653, 1]
	ωμ0_3	0.7818	[0.0193, 1]
	ωμ0_4	0.889	[0.2682, 1]
	ωμ0_5	0.8198	[0.023, 1]
	ωμ0_6	0.99	[0.3658, 1]
	ωμ0_7	0.6647	[2.1987E-8, 0.9852]
	ωμ0_8	0.8706	[0.07, 1]
Global shrinkage Hyperprior	τ	2.0617	[0.6007, 4.8382]
	η	4.026	[2.2108, 7.8915]

Supplementary Table 7. Posterior parameter estimates for the MBD model applied to all insect genera for the Permian period. Baseline origination and extinction rates (λ0 and μ0) and correlation parameters (*G*λ and *G*μ). The drivers are numbered as follows: (0) diversity of all insects through time, (1) global variation of atmospheric CO_2 through time, (2) global variation of atmospheric O_2 through time, (3) continental fragmentation through time, (4) gymnosperm diversity through time, (5) Polypodiales ferns diversity through time, (6) Spore-plants diversity through time, (7) global temperature changes through time, and (8) non-Polypodiales ferns diversity through time. Shrinkage weights (ω), based on local and global shrinkage parameters, and global shrinkage (τ). Shrinkage weights greater than 0.5 (highlighted in bold) indicate significant evidence for correlation (positive or negative depending on the respective *G*λ or *G*μ value).

Supplementary Table 8.

Parameters		Median	95% HPD Interval
Baseline rates	λ0	0.5049	[0.0559, 1.4246]
	μ0	0.2718	[1.9441E-4, 0.9503]
Correlation parameters to origination	***G*λ0_0**	**-2.1488**	**[-2.9476, -1.4322]**
	*G*λ0_1	8.3149E-5	[-0.0003, 6.1186E-4]
	***G*λ0_2**	**-7.291**	**[-9.5014, -5.1782]**
	*G*λ0_3	29.6083	[-0.7398, 54.4182]
	*G*λ0_4	-0.9792	[-4.5281, 1.2253]
	***G*λ0_5**	**-25.2355**	**[-38.3041, -10.6357]**
	*G*λ0_6	-3.1484	[-11.9014, 2.1546]
	*G*λ0_7	-0.0077	[-0.0739, 0.0804]
	***G*λ0_8**	**5.6409**	**[3.5588, 7.6032]**
Correlation parameters to extinction	***G*μ0_0**	**-0.8541**	**[-1.7276, -0.1175]**
	*G*μ0_1	-0.0004	[-0.0007, 2.2247E-5]
	*G*μ0_2	-0.3191	[-3.402, 2.8961]
	***G*μ0_3**	**-75.3476**	**[-131.2031, -35.564]**
	***G*μ0_4**	**-13.2928**	**[-16.7764, -9.8605]**
	***G*μ0_5**	**40.4752**	**[21.4145, 57.1099]**
	***G*μ0_6**	**-27.98**	**[-37.1274, -18.1788]**
	***G*μ0_7**	**0.1532**	**[0.0403, 0.2658]**
	***G*μ0_8**	**7.6078**	**[5.486, 9.7228]**
Shrinkage weights (origination)	ωλ0_0	0.936	[0.6057, 1]
	ωλ0_1	0.8425	[0.0319, 1]
	ωλ0_2	0.9859	[0.9245, 1]
	ωλ0_3	0.9838	[0.7669, 1]
	ωλ0_4	0.8215	[0.0297, 1]
	ωλ0_5	0.998	[0.9865, 1]
	ωλ0_6	0.9607	[0.2261, 1]
	ωλ0_7	0.8523	[0.0471, 1]
	ωλ0_8	0.9792	[0.8843, 1]
Shrinkage weights (extinction)	ωμ0_0	0.8378	[0.171, 1]
	ωμ0_1	0.9269	[0.3264, 1]
	ωμ0_2	0.8663	[0.0615, 1]
	ωμ0_3	0.9964	[0.9769, 1]
	ωμ0_4	0.9885	[0.9409, 1]
	ωμ0_5	0.9992	[0.9953, 1]
	ωμ0_6	0.9984	[0.992, 1]
	ωμ0_7	0.9815	[0.8566, 1]
	ωμ0_8	0.9863	[0.9278, 1]
Global shrinkage Hyperprior	τ	6.2396	[2.2152, 12.8093]
	η	4.2479	[2.2981, 6.6003]

Supplementary Table 8. Posterior parameter estimates for the MBD model applied to all insect genera for the Triassic period. Baseline origination and extinction rates (λ0 and μ0) and correlation parameters (*G*λ and *G*μ). The drivers are numbered as follows: (0) diversity of all insects through time, (1) global variation of atmospheric CO_2 through time, (2) global variation of atmospheric O_2 through time, (3) continental fragmentation through time, (4) gymnosperm diversity through time, (5) Polypodiales ferns diversity through time, (6) Spore-plants diversity through time, (7) global temperature changes through time, and (8) non-Polypodiales ferns diversity through time. Shrinkage weights (ω), based on local and global shrinkage parameters, and global shrinkage (τ). Shrinkage weights greater than 0.5 (highlighted in bold) indicate significant evidence for correlation (positive or negative depending on the respective *G*λ or *G*μ value).

Supplementary Table 9.

Parameters		Median	95% HPD Interval
Baseline rates	λ0	0.6099	[9.1542E-4, 1.2764]
	μ0	0.2623	[0.0116, 1.4412]
Correlation parameters to origination	*G*λ0_0	-0.5159	[-2.2463, 0.3403]
	*G*λ0_1	0	[-0.0007, 3.9596E-4]
	*G*λ0_2	-0.2516	[-2.665, 0.8475]
	*G*λ0_3	-1.9501	[-37.6604, 17.7567]
	*G*λ0_4	-0.0707	[-0.2089, 0.0164]
	*G*λ0_5	0.0394	[-2.5242, 3.5467]
	*G*λ0_6	-0.0044	[-0.0309, 7.8162E-3]
	*G*λ0_7	-11.8376	[-34.7947, 3.0841]
	*G*λ0_8	0.043	[-7.4429, 6.3857]
	*G*λ0_9	3.4913E-3	[-0.0567, 0.1396]
	*G*λ0_10	1.0156	[-0.9476, 4.6672]
Correlation parameters to extinction	*G*μ0_0	0.1286	[-0.763, 2.8059]
	*G*μ0_1	5.7984E-6	[-0.0004, 7.899E-4]
	*G*μ0_2	0.0127	[-1.4257, 1.8098]
	*G*μ0_3	-5.0904	[-60.3636, 11.7943]
	*G*μ0_4	0.0256	[-0.0266, 0.1909]
	*G*μ0_5	-0.4333	[-6.5751, 2.3674]
	*G*μ0_6	-0.0246	[-0.0693, 2.3785E-3]
	*G*μ0_7	7.3439	[-6.1559, 31.9604]
	*G*μ0_8	0.0258	[-6.41, 8.5797]
	*G*μ0_9	-0.0027	[-0.1224, 0.0593]
	*G*μ0_10	2.7456	[-0.7471, 6.241]
Shrinkage weights (origination)	ωλ0_0	0.3178	[2.2473E-6, 0.929]
	ωλ0_1	0.2596	[1.0519E-7, 0.952]
	ωλ0_2	0.2248	[1.3072E-6, 0.9388]
	ωλ0_3	0.2211	[1.2085E-6, 0.9105]
	ωλ0_4	0.7331	[2.0859E-6, 0.9854]
	ωλ0_5	0.1607	[2.4001E-6, 0.8863]
	ωλ0_6	0.3093	[1.2984E-6, 0.9281]
	ωλ0_7	0.3141	[3.6272E-6, 0.9233]
	ωλ0_8	0.1677	[2.3332E-7, 0.9195]
	ωλ0_9	0.1902	[5.9801E-6, 0.9101]
	ωλ0_10	0.2891	[3.3142E-8, 0.9225]
Shrinkage weights (extinction)	ωμ0_0	0.2302	[5.6282E-9, 0.9268]
	ωμ0_1	0.2579	[8.9926E-6, 0.9544]
	ωμ0_2	0.1335	[3.9108E-10, 0.8822]
	ωμ0_3	0.2961	[3.8777E-7, 0.9445]
	ωμ0_4	0.4777	[3.3949E-7, 0.9783]
	ωμ0_5	0.2562	[6.7661E-6, 0.9598]
	ωμ0_6	0.7401	[0.0326, 0.9998]
	ωμ0_7	0.2023	[1.444E-7, 0.8914]
	ωμ0_8	0.1798	[2.1794E-6, 0.929]
	ωμ0_9	0.1746	[7.9415E-7, 0.8835]
	ωμ0_10	0.5334	[1.6073E-5, 0.9621]
Global shrinkage Hyperprior	τ	0.6232	[0.1001, 1.6089]
	η	3.3294	[2.3941, 4.3133]

Supplementary Table 9. Posterior parameter estimates for the MBD model applied to all predator insects genera. Baseline origination and extinction rates (λ0 and μ0) and correlation parameters (*G*λ and *G*μ). The drivers are numbered as follows: (0) diversity of all insects through time, (1) global variation of atmospheric CO_2 through time, (2) global variation of atmospheric O_2 through time, (3) continental fragmentation through time, (4) diversity through time of non-herbivores and non-carnivores, (5) gymnosperm diversity through time, (6) diversity through time of herbivores, (7) Polypodiales ferns diversity through time, (8) Spore-plants diversity through time, (9) global temperature changes through time, and (10) non-Polypodiales ferns diversity through time. Shrinkage weights (ω), based on local and global shrinkage parameters, and global shrinkage (τ). Shrinkage weights greater than 0.5 (highlighted in bold) indicate significant evidence for correlation (positive or negative depending on the respective *G*λ or *G*μ value).

Supplementary Table 10.

Parameters		Median	95% HPD Interval
Baseline rates	λ0	0.2205	[0.0176, 0.5687]
	μ0	0.2065	[0.0195, 0.617]
Correlation parameters to origination	**Gλ0_0**	**-2.1626**	**[-3.7056, -0.8134]**
	Gλ0_1	0	[-0.0012, 9.7405E-4]
	Gλ0_2	-1.0237	[-3.2048, 0.6353]
	Gλ0_3	0.0243	[-0.0101, 0.0682]
	Gλ0_4	6.7662	[-14.8855, 47.8041]
	Gλ0_5	4.2264E-3	[-0.0501, 0.0726]
	Gλ0_6	0.8124	[-1.7495, 6.1633]
	**Gλ0_7**	**-31.4549**	**[-50.9923, -15.4651]**
	Gλ0_8	1.8732	[-5.5664, 17.8742]
	Gλ0_9	-0.0057	[-0.1712, 0.0983]
	**Gλ0_10**	**3.5198**	**[1.1187, 6.0513]**
Correlation parameters to extinction	Gμ0_0	0.3318	[-0.8962, 3.0206]
	Gμ0_1	6.5218E-4	[-0.0002, 2.9063E-3]
	Gμ0_2	-1.5815	[-4.6439, 0.4827]
	Gμ0_3	-0.0355	[-0.112, 9.1364E-3]
	Gμ0_4	-53.36	[-91.0104, 0.1368]
	**Gμ0_5**	**0.2471**	**[0.1728, 0.3227]**
	Gμ0_6	-6.8016	[-12.3045, 0.0717]
	Gμ0_7	-2.4345	[-21.1315, 11.2382]
	Gμ0_8	-0.2912	[-15.6626, 10.15]
	Gμ0_9	-0.001	[-0.1438, 0.1422]
	Gμ0_10	-0.0624	[-2.9676, 2.6919]
Shrinkage weights (origination)	ωλ0_0	0.8353	[0.4122, 1]
	ωλ0_1	0.5932	[2.022E-8, 0.9878]
	ωλ0_2	0.554	[5.7767E-8, 0.9675]
	ωλ0_3	0.5075	[2.7151E-7, 0.9603]
	ωλ0_4	0.4281	[8.4208E-9, 0.9643]
	ωλ0_5	0.357	[1.2566E-9, 0.9499]
	ωλ0_6	0.4334	[9.7502E-9, 0.9643]
	ωλ0_7	0.7296	[0.2832, 1]
	ωλ0_8	0.5714	[1.0914E-10, 0.9807]
	ωλ0_9	0.4355	[1.9141E-9, 0.9652]
	ωλ0_10	0.7256	[0.2316, 0.9999]
Shrinkage weights (extinction)	ωμ0_0	0.4992	[2.379E-9, 0.9695]
	ωμ0_1	0.9217	[0.0233, 1]
	ωμ0_2	0.6799	[0.0237, 0.9996]
	ωμ0_3	0.6314	[1.4598E-8, 0.9789]
	ωμ0_4	0.8782	[0.3465, 1]
	ωμ0_5	0.9691	[0.8655, 1]
	ωμ0_6	0.8836	[0.3195, 1]
	ωμ0_7	0.2246	[1.6247E-9, 0.925]
	ωμ0_8	0.4896	[1.0908E-7, 0.9743]
	ωμ0_9	0.4254	[3.6569E-10, 0.9662]
	ωμ0_10	0.2761	[5.3699E-8, 0.9354]
Global shrinkage Hyperprior	τ	1.2805	[0.3564, 2.7827]
	η	4.391	[2.748, 6.5907]

Supplementary Table 10. Posterior parameter estimates for the MBD model applied to all herbivorous insects genera. Baseline origination and extinction rates ($\lambda0$ and $\mu0$) and correlation parameters ($G\lambda$ and $G\mu$). The drivers are numbered as follows: (0) diversity of all insects through time, (1) global variation of atmospheric CO_2 through time, (2) global variation of atmospheric O_2 through time, (3) diversity through time of predators, (4) continental fragmentation through time, (5) diversity through time of non-herbivores and non-carnivores, (6) gymnosperm diversity through time, (7) Polypodiales ferns diversity through time, (8) Spore-plants diversity through time, (9) global temperature changes through time, and (10) non-Polypodiales ferns diversity through time. Shrinkage weights (ω), based on local and global shrinkage parameters, and global shrinkage (τ). Shrinkage weights greater than 0.5 (highlighted in bold) indicate significant evidence for correlation (positive or negative depending on the respective $G\lambda$ or $G\mu$ value).

Supplementary Table 11.

Parameters		Median	95% HPD Interval
Baseline rates	λ0	0.0784	[3.9364E-3, 0.3066]
	μ0	0.1959	[0.0329, 0.4548]
Correlation parameters to origination	*G*λ0_0	-0.0217	[-2.1037, 1.3936]
	*G*λ0_1	0	[-0.0005, 4.2434E-4]
	*G*λ0_2	-0.0598	[-1.6856, 1.2288]
	*G*λ0_3	1.0112E-3	[-0.0332, 0.0514]
	*G*λ0_4	-4.4934	[-48.7644, 10.9238]
	*G*λ0_5	0.06	[-2.2819, 3.4306]
	*G*λ0_6	3.1118E-3	[-0.008, 0.0424]
	*G*λ0_7	0.1761	[-12.9402, 15.8065]
	*G*λ0_8	-0.2496	[-17.9503, 5.9614]
	*G*λ0_9	-0.0063	[-0.1351, 0.0485]
	*G*λ0_10	1.5893	[-1.0279, 7.2546]
Correlation parameters to extinction	*G*μ0_0	0.0915	[-0.6697, 2.8568]
	*G*μ0_1	0	[-0.0007, 4.8008E-4]
	*G*μ0_2	-0.0442	[-1.6793, 0.7757]
	*G*μ0_3	0	[-0.0429, 0.04]
	*G*μ0_4	0.4442	[-14.4759, 33.0578]
	*G*μ0_5	-0.0221	[-2.7488, 2.2633]
	*G*μ0_6	-0.0037	[-0.0359, 7.7561E-3]
	*G*μ0_7	1.1439	[-8.5253, 20.2291]
	*G*μ0_8	0.0385	[-6.0751, 7.0477]
	*G*μ0_9	-0.0017	[-0.0976, 0.0512]
	*G*μ0_10	-1.1354	[-6.0459, 0.6731]
Shrinkage weights (origination)	ωλ0_0	0.0812	[1.1673E-6, 0.8825]
	ωλ0_1	0.1009	[1.0987E-6, 0.9145]
	ωλ0_2	0.0716	[8.7489E-8, 0.8274]
	ωλ0_3	0.088	[6.2246E-8, 0.8245]
	ωλ0_4	0.2069	[4.5203E-7, 0.9352]
	ωλ0_5	0.0812	[5.1209E-7, 0.8165]
	ωλ0_6	0.1753	[5.0527E-8, 0.9304]
	ωλ0_7	0.0417	[1.92E-8, 0.6428]
	ωλ0_8	0.1234	[4.973E-6, 0.9532]
	ωλ0_9	0.1114	[1.841E-7, 0.9359]
	ωλ0_10	0.332	[1.5929E-7, 0.9509]
Shrinkage weights (extinction)	ωμ0_0	0.1053	[5.114E-7, 0.8878]
	ωμ0_1	0.0887	[2.3165E-8, 0.9554]
	ωμ0_2	0.0702	[5.9168E-7, 0.7715]
	ωμ0_3	0.0764	[3.5389E-10, 0.8351]
	ωμ0_4	0.0747	[7.0403E-8, 0.8226]
	ωμ0_5	0.067	[3.2155E-8, 0.7766]
	ωμ0_6	0.19	[1.2513E-6, 0.9219]
	ωμ0_7	0.0575	[6.0692E-8, 0.7098]
	ωμ0_8	0.0895	[2.1435E-8, 0.8672]
	ωμ0_9	0.0763	[3.1846E-8, 0.8719]
	ωμ0_10	0.2349	[2.2403E-7, 0.9188]
Global shrinkage Hyperprior	τ	0.364	[0.0281, 0.9951]
	η	6.4594	[4.5576, 8.4123]

Supplementary Table 11. Posterior parameter estimates for the MBD model applied to all non-herbivores and non-predators genera. Baseline origination and extinction rates (λ0 and μ0) and correlation parameters (*G*λ and *G*μ). The drivers are numbered as follows: (0) diversity of all insects through time, (1) global variation of atmospheric CO_2 through time, (2) global variation of atmospheric O_2 through time, (3) diversity through time of predators, (4) continental fragmentation through time, (5) gymnosperm diversity through time, (6) diversity through time of herbivores, (7) Polypodiales ferns diversity through time, (8) Spore-plants diversity through time, (9) global temperature changes through time, and (10) non-Polypodiales ferns diversity through time. Shrinkage weights (ω), based on local and global shrinkage parameters, and global shrinkage (τ). Shrinkage weights greater than 0.5 (highlighted in bold) indicate significant evidence for correlation (positive or negative depending on the respective *G*λ or *G*μ value).

Supplementary Table 12.

Parameters		Median	95% HPD Interval
Baseline speciation	λ0	0.0386	[0.0271, 0.0551]
	λ1	0.1462	[0.0262, 0.1966]
	λ2	0.058	[0.0237, 0.1231]
	λ3	0.1453	[0.0937, 0.2008]
Baseline extinction	μ0	0.0471	[0.0263, 0.0681]
	μ1	0.1584	[0.0765, 0.2158]
	μ2	0.0855	[0.0274, 0.1857]
	μ4	0.0941	[0.0381, 0.1365]
Negative or positive interaction over herbivores origination	*g*λ0_0	0	[-0.0401, 0.0538]
	*g*λ0_1	0	[-0.06, 0.1588]
	***g*λ0_2**	**-0.2637**	**[-0.3, -0.1609]**
	***g*λ0_3**	**-0.2278**	**[-0.3, -0.1334]**
Negative or positive interaction over herbivores extinction	***g*μ0_0**	**0.0514**	**[-0, 0.1106]**
	***g*μ0_1**	**-0.1434**	**[-0.2789, 0]**
	***g*μ0_2**	**0.2724**	**[0.1937, 0.3]**
	***g*μ0_3**	**0.0988**	**[0.0286, 0.1961]**
Negative or positive interaction over predators origination	*g*λ1_0	0	[-0.1069, 4.6815E-3]
	*g*λ1_1	0	[-0.0058, 0.2552]
	*g*λ1_2	0	[-0.1345, 0.1299]
	*g*λ1_3	0	[-0.2318, 2.8857E-3]
Negative or positive interaction over predators extinction	*g*μ1_0	0	[-0.0003, 0.01]
	*g*μ1_1	0	[-0.0003, 0.059]
	*g*μ1_2	0	[-0.1057, 0.0218]
	*g*μ1_3	0	[-0.0152, 8.5417E-4]
Negative or positive interaction over generalists origination	*g*λ2_0	0	[-0.1079, 7.0951E-3]
	*g*λ2_1	0	[-0.0221, 0.2876]
	*g*λ2_2	0	[-0.2884, 0.1817]
	***g*λ2_3**	**-0.2145**	**[-0.3, -0.0704]**
Negative or positive interaction over generalists extinction	*g*μ2_0	0	[-0.0535, 8.1465E-3]
	*g*μ2_1	0	[-0.1297, 0.0544]
	*g*μ2_2	0.2131	[-0, 0.295]
	*g*μ2_3	0.1026	[-0, 0.2777]
Negative or positive interaction over detritivores/fungivore origination	*g*λ3_0	6.8037E-3	[-0, 0.0133]
	*g*λ3_1	0	[-0, 0.0376]
	*g*λ3_2	0	[-0.1478, 0.0195]
	*g*λ3_3	0	[-0, 0]
Negative or positive interaction over detritivores/fungivore extinction	*g*μ3_0	0	[-0.0144, 0.0594]
	*g*μ3_1	0	[-0.182, 2.3447E-3]
	*g*μ3_2	0.0472	[-0, 0.2833]
	*g*μ3_3	0	[0, 0.0569]
Hyperprior	η0	0.2724	[1.1861E-4, 0.6102]
	η1	0.7617	[0.3685, 0.9998]
	η2	0.4982	[0.1266, 0.8478]
	η3	0.6612	[0.3031, 0.9681]
	ηR	11.2084	[4.4577, 20.4883]

Supplementary Table 12. Posterior parameter estimates for the MCDD model. The clades are numbered as follows: (0) Herbivores, (1) Predators, (2) Generalists, (3) Detritivores/Fungivores. Baseline origination represents the origination rate for a clade in absence of diversity dependence. $g\lambda0\_0$ denotes for diversity dependence in the clade 0. $g\lambda0\_1 > 0$ indicates the diversity of clade 1 negatively correlates with origination of clade 0 (increasing diversity of clade 1 correlates with low origination of clade 0). $g\lambda0\_1 < 0$ indicates the diversity of clade 1 positively correlates with origination of clade 0 (increasing diversity of clade 1 correlates with high origination of clade 0). Bold values are significant correlations.

Supplementary Table 13.

Parameters		Median	95% HPD Interval
Baseline speciation	λ0	0.0343	[0.0259, 0.0489]
	λ1	0.1492	[0.0278, 0.1875]
	λ2	0.1553	[0.1043, 0.2105]
Baseline extinction	μ0	0.0347	[0.019, 0.0559]
	μ1	0.1566	[0.0633, 0.2097]
	μ2	0.0768	[0.0254, 0.1376]
Negative or positive interaction over herbivores origination	*g*λ0_0	0	[-0.0609, 0.0501]
	*g*λ0_1	0	[-0.0385, 0.2179]
	***g*λ0_2**	**-0.2649**	**[-0.3, -0.1786]**
Negative or positive interaction over herbivores extinction	***g*μ0_0**	**0.0789**	**[-0, 0.1278]**
	***g*μ0_1**	**-0.2054**	**[-0.2942, -0]**
	***g*μ0_2**	**0.1718**	**[0.0881, 0.2993]**
Negative or positive interaction over predators origination	*g*λ1_0	0	[-0.102, 8.1915E-3]
	*g*λ1_1	0	[-0.0078, 0.2542]
	*g*λ1_2	0	[-0.2275, 2.5244E-3]
Negative or positive interaction over predators extinction	*g*μ1_0	0	[-0.0003, 0.0137]
	*g*μ1_1	0	[-0.0039, 0.0561]
	*g*μ1_2	0	[-0.0185, -0]
Negative or positive interaction over Others origination	*g*λ2_0	0	[-0, 0.011]
	***g*λ2_1**	**0.0118**	**[0, 0.0276]**
	*g*λ2_2	0	[-0.0003, 7.1998E-4]
Negative or positive interaction over Others extinction	*g*μ2_0	0	[-0.0152, 0.0704]
	*g*μ2_1	0	[-0.204, 9.9466E-3]
	***g*μ2_2**	**0.0302**	**[0, 0.1386]**
Hyperprior	η0	0.3326	[3.2629E-5, 0.7215]
	η1	0.7842	[0.3342, 0.9999]
	η2	0.6385	[0.2553, 0.9706]
	ηR	11.3038	[3.5165, 22.3452]

Supplementary Table 13. Posterior parameter estimates for the MCDD model. The clades are numbered as follows: (0) Herbivores, (1) Predators, (2) Others (=Generalists plus Detritivores/ Fungivores). Baseline origination represents the origination rate for a clade in absence of diversity dependence. *g*λ0_0 denotes for diversity dependence in the clade 0. *g*λ0_1 > 0 indicates the diversity of clade 1 negatively correlates with origination of clade 0 (increasing diversity of clade 1 correlates with low origination of clade 0). *g*λ0_1 < 0 indicates the diversity of clade 1 positively correlates with origination of clade 0 (increasing diversity of clade 1 correlates with high origination of clade 0). Bold values are significant correlations.

Clades	Genus		Family	
	**Nb of occurences**	**Nb of genus in PyRate**	**Nb of occurrences**	**Nb of family in PyRate**
Coleoptera	1010	205	X	X
Holometabola	3122	435	3027	84
Palaeoptera	1439	180	1016	75
Mecoptera	1626	121	X	X
Hemiptera	1771	266	X	X
Acercaria	2849	321	2865	64
Orthoptera + Titanoptera	983	99	X	X
Polyneoptera	6655	726	6484	145
Palaeodictyopteroidea	512	89	X	X
Genus	14789	1784	X	X
Family	14483	418	X	X
Family without singleton	14483	325	X	X

Supplementary Table 14. Composition of each dataset. The number of occurrences *per* analysis is detailed for each clades.
