# Supplementary material for: Multiple drivers and lineage-specific insect extinctions during the Permo–Triassic
Source: Nat Commun. 2022 Dec 6;13:7512. doi: 10.1038/s41467-022-35284-4 (PMC9726944; doi:10.1038/s41467-022-35284-4)
Supplement: Supplementary file 3 — Description of Additional Supplementary Files [file 41467_2022_35284_MOESM3_ESM.pdf]

Description of Additional Supplementary Files

Supplementary Data 1:

All datasets used for the RJMCMC and BDCS analyses: it includes the dataset for each insect clade analyzed at the family and genus level, plus the datasets used for higher systematic clades (e.g., Polyneoptera, Holometabola). The content of each spreadsheet is detailed here:

-Fam_level: occurrences of fossil insects compiled at the family level
-Genus_level: occurrences of fossil insects compiled at the genus level
-Coleoptera_genus_level: occurrences of Coleoptera compiled at the genus level
-Mecoptera_genus_level: occurrences of Mecoptera compiled at the genus level
-Hemiptera_genus_level: occurrences of Hemiptera compiled at the genus level
-Orthoptera_Titanoptera_genus_le: occurrences of Orthoptera and Titanoptera compiled at the genus level
-Odonatoptera_genus_level: occurrences of Odonatoptera compiled at the genus level
-Palaeodictyopteroidea_genus_lev: occurrences of Palaeodictyopteroidea compiled at the genus level
-Acercaria_genus_level: occurrences of Acercaria compiled at the genus level
-Holometabola_genus_level: occurrences of Holometabola compiled at the genus level
-Palaeoptera_genus_level: occurrences of Palaeoptera compiled at the genus level
-Polyneoptera_genus_level: occurrences of Polyneoptera compiled at the genus level
-Acercaria_familiy_level: occurrences of Acercaria compiled at the family level
-Holometabola_family_level: occurrences of Holometabola compiled at the family level
-Palaeoptera_family_level: occurrences of Palaeoptera compiled at the family level
-Polyneoptera_family_level: occurrences of Polyneoptera compiled at the family level
-Family_family_level: occurrences of Family compiled at the family level

All the guilds assignations at the genus level used for the MCDD analyses (spreadsheet: MCDD_assignation)

All the spreadsheets of the time-continuous paleo-environmental variables used for the MBD model in PyRate are detailed:

-CO2: fluctuation of CO2 through the Permo-Triassic interval
-O2: fluctuation of O2 through the Permo-Triassic interval
-Continental_fragmentation: index of continental fragmentation through the Permo-Triassic interval
-Temperature: fluctuation of temperature through the Permo-Triassic interval
-Spore_plants: relative abundance of spore plants through the Permo-Triassic interval

-Polypodiales: relative abundance of polypodiales ferns through the Permo-Triassic interval

-Non_polypodiales: relative abundance of non polypodiales ferns through the Permo-Triassic interval

-Gymnosperms: relative abundance of gymnosperms through the Permo-Triassic interval

All the references used to construct the different datasets (spreadsheet: References)

Supplementary Code:

-A command list of the Bayesian analyses used to perform analyses using PyRate
