# Supplementary material for: Multiple drivers and lineage-specific insect extinctions during the Permo–Triassic
Source: Nat Commun. 2022 Dec 6;13:7512. doi: 10.1038/s41467-022-35284-4 (PMC9726944; doi:10.1038/s41467-022-35284-4)
Supplement: Supplementary file 6 — Reporting Summary [file 41467_2022_35284_MOESM6_ESM.pdf]

nature portfolio

Corresponding author(s): Corentin Jouault, Fabien L. Condamine

Last updated by author(s): 13/11/22

Reporting Summary

Nature Portfolio wishes to improve the reproducibility of the work that we publish. This form provides structure for consistency and transparency in reporting. For further information on Nature Portfolio policies, see our Editorial Policies and the Editorial Policy Checklist.

Statistics

For all statistical analyses, confirm that the following items are present in the figure legend, table legend, main text, or Methods section.

n/a	Confirmed	
☐	☒	The exact sample size (n) for each experimental group/condition, given as a discrete number and unit of measurement
☒	☐	A statement on whether measurements were taken from distinct samples or whether the same sample was measured repeatedly
☒	☐	The statistical test(s) used AND whether they are one- or two-sided<br>*Only common tests should be described solely by name; describe more complex techniques in the Methods section.*
☐	☒	A description of all covariates tested
☒	☐	A description of any assumptions or corrections, such as tests of normality and adjustment for multiple comparisons
☐	☒	A full description of the statistical parameters including central tendency (e.g. means) or other basic estimates (e.g. regression coefficient) AND variation (e.g. standard deviation) or associated estimates of uncertainty (e.g. confidence intervals)
☒	☐	For null hypothesis testing, the test statistic (e.g. F, t, r) with confidence intervals, effect sizes, degrees of freedom and P value noted<br>*Give P values as exact values whenever suitable.*
☐	☒	For Bayesian analysis, information on the choice of priors and Markov chain Monte Carlo settings
☒	☐	For hierarchical and complex designs, identification of the appropriate level for tests and full reporting of outcomes
☒	☐	Estimates of effect sizes (e.g. Cohen's d, Pearson's r), indicating how they were calculated

Our web collection on statistics for biologists contains articles on many of the points above.

Software and code

Policy information about availability of computer code

Data collection	We collected fossil data for the insects for which specimen occurrences are available in Paleobiology Database (https://paleobiodb.org/), cleaned the dataset with our expertise, and densely complemented the dataset using the bibliography. No dedicated software was used for data collection.
Data analysis	We used PyRate v.3 (https://github.com/dsilvestro/PyRate) to perform all analyses. PyRate is a Bayesian model to estimate origination, extinction and preservation rates from fossil occurrence data. We used Tracer v1.7.2 (https://github.com/beast-dev/tracer/releases) to monitor chain mixing and the effective sample size of each analyze.

For manuscripts utilizing custom algorithms or software that are central to the research but not yet described in published literature, software must be made available to editors and reviewers. We strongly encourage code deposition in a community repository (e.g. GitHub). See the Nature Portfolio guidelines for submitting code & software for further information.

Data

Policy information about availability of data

All manuscripts must include a data availability statement. This statement should provide the following information, where applicable:

- Accession codes, unique identifiers, or web links for publicly available datasets
- A description of any restrictions on data availability
- For clinical datasets or third party data, please ensure that the statement adheres to our policy

We collected fossil data for the insects for which specimen occurrences are available in Paleobiology Database (https://paleobiodb.org/), cleaned the dataset with

our expertise, and densely complemented the dataset using the bibliography. The data generated in this study are provided in the Supplementary Data. The datasets used for the RJMCMC and BDCS analyses, all the guilds assignations for the MCDD analyses, all the paleo-environmental variables used for the MBD model, and all the references used to construct the different datasets can be found in Supplementary Data and have been deposited in the Figshare digital data repository (https://doi.org/10.6084/m9.figshare.c.6296196.v1). Source data are provided as a Source Data file.

Human research participants

Policy information about studies involving human research participants and Sex and Gender in Research.

Reporting on sex and gender	N/A
Population characteristics	N/A
Recruitment	N/A
Ethics oversight	N/A

Note that full information on the approval of the study protocol must also be provided in the manuscript.

Field-specific reporting

Please select the one below that is the best fit for your research. If you are not sure, read the appropriate sections before making your selection.

☐ Life sciences ☐ Behavioural & social sciences ☒ Ecological, evolutionary & environmental sciences

For a reference copy of the document with all sections, see nature.com/documents/nr-reporting-summary-flat.pdf

Ecological, evolutionary & environmental sciences study design

All studies must disclose on these points even when the disclosure is negative.

Study description	We analysed the temporal dynamics – modelling the origination and the extinction – of insects during the Permo-Triassic and investigated which biotic (including clades' interactions) and abiotic factors best explained these processes using a Bayesian fossil-based approach explicitly modelling preservation rates as well as times of origination and extinction of each genus.
Research sample	Our sample is exhaustive in regard to the literature and includes all the fossil insect occurrences known for the Permo-Triassic time interval. This allows for drawing the most accurate picture of the insect dynamics during this time interval. These data combine all the populations of fossil insects geographically distant (published in the literature) in one dataset to estimate the impact of putative extinction events at a global scale. The original dataset was downloaded from the publicly available Paleobiology Database (https://paleobiodb.org) and is composed of occurrences i.e., specimens originating from a given stratigraphic horizon assigned to a given taxon.
Sampling strategy	No sampling strategy was applied for this study as we used all the fossil insect occurrences known for the Permo-Triassic time interval. The sample size was determined by availability of fossil data for insects. We used all available data in our study. The sample size used in our study corresponds to most of published fossil-based diversification studies. Moreover, the extent of speciation and extinction rates heterogeneity across insects as inferred using PyRate is sufficiently elevated as to enable us to detect drivers of diversification and/or extinction.
Data collection	C.J compiled all species-level fossil occurrences of insects using https://paleobiodb.org/ (PBDB) as a starting point (downloaded October 12, 2021). The dataset was cleaned of synonyms, outdated combinations, nomina dubia, and other erroneous and doubtful records, based on revisions provided in the literature and/or on the expertise of the authors. After correction including data addition from the literature, our dataset was composed of 3,636 species (1,784 genera, and 418 families) for 17,250 occurrences resulting from an in-depth study and curation of the entire bibliography of fossil insects, spanning from the Asselian (lowermost Permian) to the Rhaetian (uppermost Triassic). The data compilation was carried out manually by C.J in a spreadsheet where each occurrence (i.e., specimen originating from a given stratigraphic horizon assigned to a given taxon) was placed in a distinct spreadsheet line.
Timing and spatial scale	Timing scale (scope) of the study is the Permian and Triassic. The spatial scale is global, although the sampling can be considered as slightly biased with the Northern Hemisphere being more sampled (more fossil localities). Our dataset encompasses all the occurrences between the first formal description of fossil insects from the Permian period and the data of the data extraction from https://paleobiodb.org/ October 12, 2021. No periodicity or frequency was applied as we used all the known fossil occurrences. No collection periods (i.e., gap) were recorded as the description of fossil insects is a relatively continuous process.
Data exclusions	We excluded taxa that are nomina dubia and ichnospecies, which cannot be attributed to a genus or family of insects.
Reproducibility	Our data are not experimental and experiments were thus not replicated. However, we used multiple distinct statistical tests (e.g. environment-dependent diversificaiton models, multivariate birth-death models) and all approaches yielded concordant results.
Randomization	We did not perform an experiment and there was thus no group allocation. We used all insect taxa for which fossil occurrence data were available. For analyses of clade interactions, there was further group partitioning of data by associating a taxon to a diet when

	possible.
Blinding	Blinding was not relevant to our study, because all available data were used and no sampling bias can be recorded. Moreover, our study did not perform an experiment in which information may influence the final result.

Did the study involve field work? ☐ Yes ☒ No

Reporting for specific materials, systems and methods

We require information from authors about some types of materials, experimental systems and methods used in many studies. Here, indicate whether each material, system or method listed is relevant to your study. If you are not sure if a list item applies to your research, read the appropriate section before selecting a response.

Materials & experimental systems

n/a	Involved in the study
☒	☐ Antibodies
☒	☐ Eukaryotic cell lines
☐	☒ Palaeontology and archaeology
☒	☐ Animals and other organisms
☒	☐ Clinical data
☒	☐ Dual use research of concern

Methods

n/a	Involved in the study
☒	☐ ChIP-seq
☒	☐ Flow cytometry
☒	☐ MRI-based neuroimaging

Palaeontology and Archaeology

Specimen provenance	No new specimens are provided. The fossil datasets are made through a compilation of the fossil data available in Paleobiology Database (https://paleobiodb.org/).
Specimen deposition	All specimens have already been deposited into Museum institutions, and in online database such as Paleobiology Database (https://paleobiodb.org/).
Dating methods	No new dates are provided. Ages of fossil occurrences come from the stratigraphic data bearing the fossils. Chronostratigraphic data come from the International Commission on Stratigraphy and sensu ,Gradstein, F.M, Ogg, J.G., Schmitz, M.D., et al., 2012, The Geologic Time Scale 2012: Boston, USA, Elsevier, https://doi.org/10.1016/B978-0-444-59425-9.00004-4.

☒ Tick this box to confirm that the raw and calibrated dates are available in the paper or in Supplementary Information.

Ethics oversight	NA

Note that full information on the approval of the study protocol must also be provided in the manuscript.
